# Synthesis, Anticancer Activity, and *In Silico* Modeling of Alkylsulfonyl Benzimidazole Derivatives: Unveiling Potent Bcl-2 Inhibitors for Breast Cancer

**DOI:** 10.1021/acsomega.3c09411

**Published:** 2024-02-14

**Authors:** Yemna Abbade, Mehmet Murat Kisla, Mohammed Al-Kassim Hassan, Ismail Celik, Tugba Somay Dogan, Pelin Mutlu, Zeynep Ates-Alagoz

**Affiliations:** † Department of Pharmaceutical Chemistry, Faculty of Pharmacy, Ankara University, 06100 Ankara, Turkey; ‡ Graduate School of Health Sciences, Ankara University, 06110 Ankara, Turkey; § Department of Pharmaceutical and Medicinal Chemistry, Faculty of Pharmaceutical Sciences, Bayero University, P.M.B 3011 Kano, Nigeria; ∥ Department of Pharmaceutical Chemistry, Faculty of Pharmacy, Erciyes University, 38039 Kayseri, Turkey; ⊥ Central Laboratory, Molecular Biology and Biotechnology R&D Center, Middle East Technical University, 06800 Ankara, Turkey; # Department of Biotechnology, Biotechnology Institute, Ankara University, 06135 Ankara, Turkey

## Abstract

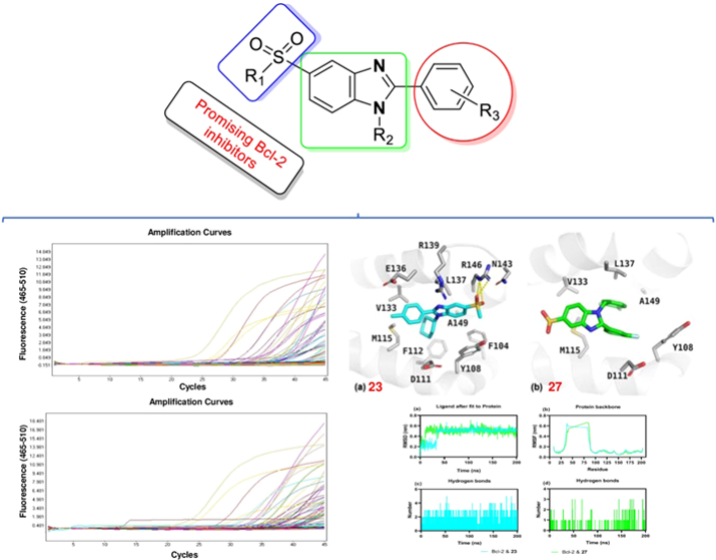

A series of alkylsulfonyl 1*H*-benzo[*d*]imidazole derivatives were synthesized and evaluated for anticancer activity against human breast cancer cells, MCF-7 *in vitro*. The cytotoxic potential was determined using the xCELLigence real-time cell analysis, and expression levels of genes related to microtubule organization, tumor suppression, apoptosis, cell cycle, and proliferation were examined by quantitative real-time polymerase chain reaction. Molecular docking against Bcl-2 was carried out using AutoDock Vina, while ADME studies were performed to predict the physicochemical and drug-likeness properties of the synthesized compounds. The results revealed that compounds **23** and **27** were the most potent cytotoxic derivatives against MCF-7 cells. Gene expression analysis showed that BCL-2 was the most prominent gene studied. Treatment of MCF-7 cells with compounds **23** and **27** resulted in significant downregulation of the BCL-2 gene, with fold changes of 128 and 256, respectively. Docking analysis predicted a strong interaction between the compounds and the target protein. Interestingly, all of the compounds exhibit a higher binding affinity toward Bcl-2 than the standard drug (compound **27** vina score = −9.6 kcal/mol, vincristine = −6.7 kcal/mol). Molecular dynamics simulations of compounds **23** and **27** showed a permanent stabilization in the binding site of Bcl-2 for 200 ns. Based on Lipinski and Veber’s filters, all synthesized compounds displayed drug-like characteristics. These findings suggest that compounds **23** and **27** were the most promising cytotoxic compounds and downregulated the expression of the BCL-2 gene. These derivatives could be further explored as potential candidates for the treatment of breast cancer.

## 1 Introduction

**Figure 1:**
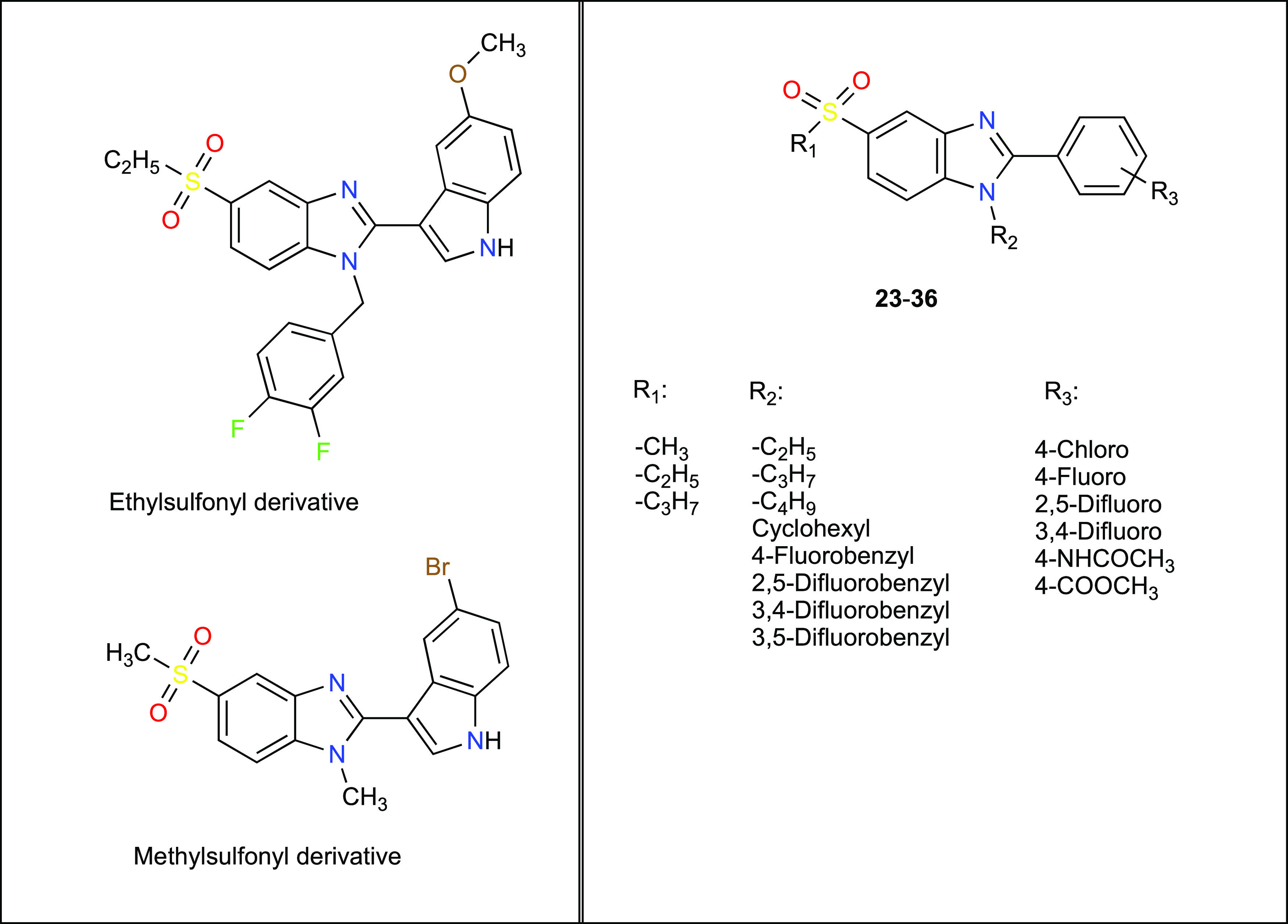
Chemical structures of previously synthesized benzimidazole derivatives and newly synthesized novel alkylsulfonyl benzimidazoles.

Globally, cancer is a significant cause of mortality and morbidity, and its universal spread is independent of the regional human development index.^1^ The disease burden is projected to reach 28.4 million cases by 2040, representing a 47% increase from the 19.3 million cases recorded in 2020.^1^ This alarming projection underscores the need to search for newer, safer, and more efficacious antineoplastic agents that target the key hallmarks of cancer. The circumvention of apoptosis is one of the essential features of cancer development and progression.^2^ Although malignant cells are well equipped with apoptosis machinery, they tend to develop strategies to block or knock off the programmed cell death mechanisms.^3^ This knock-off effect occurs when there is a defect or compromise in the apoptosis mechanism, which promotes cancer cell growth and survival.^4^ The Bcl-2 family of proteins, comprising pro- and antiapoptotic subcategories, regulates the cascade of events that culminate in apoptosis. Members of the antiapoptotic proteins include Bcl-2, Bcl-xL, Mcl-1, Bcl-w, and Bfl-1, while their pro-apoptotic counterparts include the effector proteins (Bax, Bak, and Bok) and BH3-only subfamily of proteins.^3^ The antiapoptotic Bcl-2 member proteins are often overexpressed in various malignant cells postdiagnosis and after developing resistance to cancer therapeutics.^5,6^ Their overexpression may drive carcinogenesis.^7^ Hence, targeting Bcl-2 protein is an important strategy in the development of cancer therapeutics. Bcl-2 plays a significant role in angiogenesis and growth of cancer cells.^8^ It is involved in processes that promote cellular growth and survival that antagonize the Bcl-2 family of proteins, which in turn inhibits apoptosis.^9^ Overexpression of Bcl-2 is involved in the progression of various cancers.^10^ The phosphorylation and elevated expression of Bcl-2 regulates cell survival, proliferation, DNA repair, cell cycle, and tumorigenesis. Furthermore, Bcl-2 has also been linked with several human cancers.^11,12^ Due to the pivotal role of Bcl-2 in regulating/inhibiting apoptosis, the Bcl-2 protein is a strategic and potent target for the development of novel anticancer agents for various cancers.^4^ Hence, Bcl-2 inhibitors are considered remarkable therapeutic targets for cancer therapy. Several novel molecules have been utilized as Bcl-2 inhibitors including benzimidazoles.^13^ The benzimdazole represents an important class of novel heterocyclic aromatic compounds, primarily formed by the fusion of benzene and imidazole ring systems. They act as pharmacophores for natural and synthetic molecules, with diverse applications in medicinal chemistry.^13^ Benzimidazole scaffold and their derivatives have broad-spectrum therapeutic applications, including anticancer, antiviral, anthelminthic, antiprotozoal, anti-inflammatory, analgesic, antihypertensive, and antidiabetic effects.^14−16^ Due to the diverse pharmacological activities and great prospects of this scaffold in drug discovery, benzimidazole and its derivatives have been widely studied to unravel their novelty in tackling challenging diseases, including cancer. Several benzimidazole-based synthetic compounds have been evaluated and reported to exhibit remarkable anticancer activities against many cancer cells.^17^ In the screening for anticancer therapeutics, the inhibition of Bcl-2 protein is a key target.^18^ Some benzimidazole and substituted derivatives have been described as novel tyrosine kinase and/or Bcl-2 inhibitors. These compounds demonstrate impressive anticancer activity by acting on these pathways.^19,20^ Previously, we designed and synthesized novel indole-benzimidazole derivatives bearing an ethylsulfonyl moiety and reported their anticancer effects in estrogen-responsive MCF-7 breast cancer cells.^21^ These compounds also displayed antiestrogenic activity, which provided additional insights into their potential use in breast cancer, and their interaction with molecular targets suggested that they bind to multiple genes and pathways. Furthermore, structure–activity relationship (SAR) studies suggest that the presence of a fluoro-substituted benzyl group at the R_1_ position (Figure 1) has higher anticancer activity. In a related article, the anticancer activity of scaffolds possessing methylsulfonyl moiety against MCF-7^22^ and HeLa^23^ cancer cells has also been reported. To gain further insights into their anticancer activities, our research group compared methylsulfonyl indole-benzimidazole derivatives with their ethylsulfonyl counterparts.^24^ We reported both categories of compounds exhibited anticancer effects and potential estrogen receptor modulatory actions, alluding these effects to the effective substitutions at the R_1_ and R_2_ positions of the ring and sulfonyl side chain modifications, respectively. Based on the aforementioned study, a potent methylsulfonyl-substituted benzimidazole was unraveled,^24^ as shown in Figure 1. The present study is a follow-up to our previous work on the benzimidazole scaffold wherein our goal is to obtain new synthetic compounds with profound activities as promising therapeutics. Herein, we aimed to synthesize novel benzimidazole derivatives bearing alkylsulfonyl and substituted benzene side chains and evaluate their anticancer activity in human breast cancer cell line MCF-7 *in vitro*. Furthermore, the structural characterization, ADME properties, and molecular docking studies of the compounds against Bcl-2 and other apoptotic pathway-related genes were investigated.

## 2 Experimental Section

### 2.1 Chemicals and Reagents

All chemicals, including solvents and reagents, were purchased from Sigma-Aldrich, and used for experiments without additional purification. All synthetic processes were performed under ambient conditions at specified temperatures.

### 2.2 Physical Measurements

The melting points of the compounds were determined in open capillary tubes using a Buchi B540 melting point apparatus without correction. The progress of the reactions was monitored using silica gel thin-layer chromatography (TLC) Kieselgel 60 F_254_ precoated plates (Merck, Germany) and visualized under a UV lamp at a wavelength of 254 nm. NMR spectra recorded in DMSO-*d*_6_ were obtained on a Varian Mercury FT-NMR spectrometer (Varian, Inc., Palo Alto, California) using tetramethylsilane as an internal standard. Spectra were acquired at 400 or 500 MHz for ^1^H NMR, and at 125 or 100 MHz for ^13^C NMR. The values for chemical shifts (δ values) and coupling constants (*J* values) are provided in ppm and Hz, respectively. Mass analyses were performed on a Waters ZQ Micromass liquid chromatography–mass spectrometry (LC–MS) spectrometer (Waters Co., Milford, MA) using electrospray ionization. Elemental analyses were performed using Leco CHNS 932 Elemental Analyzer (Leco-932, St. Joseph, MI).

### 2.3 Chemical Synthesis

The general synthesis of the compounds is based on our previous work.^21,24^ The synthesis of 1-chloro-4-(alkylsulfonyl)-benzene (**1**–**3**) started with the alkylation of the commercially available 4-chlorobenzenesulfonyl chloride. Nitration of **1**–**3** at position 2 in the presence of potassium nitrate and sulfuric acid yields 1-chloro-4-(alkylsulfonyl)–2-nitrobenzene (**4**–**6**). Aromatic nucleophilic substitution of the chlorine atom of **4**–**6** with an appropriate amine yields the corresponding 1-substituted-amino-4-(methylsulfonyl)–2-nitrobenzene (**7**–**14**), followed by the reduction of the nitro group to give **15**–**22**. The physicochemical data for these intermediates have been reported in our earlier publications.^21,24^

#### 2.3.1 General Methods for the Synthesis of Alkylsulfonyl 1*H*-Benzo[*d*]imidazole Derivatives (**23**–**36**)

A solution of substituted benzaldehyde in ethanol (20 mL) was added to a solution of sodium metabisulfite in water (20 mL). The mixture was stirred in an ice bath to produce a white precipitate, which was then filtered and dried (mp decom. >300 °C). The sodium metabisulfite adduct of benzaldehyde and the appropriate 1,2-phenylenediamine in dimethylformamide (5 mL) was heated at 130 °C for 4 h. The reaction mixture was poured onto ice, filtered, and purified by column chromatography (CC). Crystallization from methanol produced compounds **23**–**36**.^25^

##### 2-(4-Chlorophenyl)–1-cyclohexyl-5-(methylsulfonyl)–1*H*-benzo[*d*]imidazole (**23**)

Compound **23** was synthesized based on the general method, beginning with N^1^-cyclohexyl-4-(methylsulfonyl) benzene-1,2-diamine (0.97 mmol, 0.243 g) and sodium (4-chlorophenyl)-hydroxymethanesulfonate (0.97 mmol, 0.238 g). The resulting product was purified by CC using ethyl acetate/hexane (1:1) solvent system (0.071 g, 20.2% yield). M.P: 239.2 °C, MS (ESI^+^) [M + H]^+^ (%): 389.56 (100%). ^1^H NMR (400 MHz, DMSO-*d*_6_): δ ppm 1.42–1.22 (m, 3H, cyclohexyl), 1.63 (d, *J* = 11.6 Hz, 1H, cyclohexyl), 1.84 (d, *J* = 12.8 Hz, 2H, cyclohexyl), 1.94 (d, *J* = 11.2 Hz, 2H, cyclohexyl),, 2.23–2.32 (m, 2H, cyclohexyl), 3.24 (s, 3H, CH_3_), 4.23–4.29 (m, 1H, cyclohexyl), 7.68–7.73 (m, 4H), 7.80 (dd, *J* = 8.4 Hz, *J* = 2 Hz, 1H), 8.18 (d, *J* = 8.8 Hz, 1H), 8.23 (d, *J* = 2 Hz, 1H). ^13^C NMR (DMSO-*d*_6_): δ ppm 24.27, 25.38, 30. 44, 44.19, 57.10, 113.99, 119.13, 120. 55, 128.80, 128.98, 131.30, 134. 47, 135.14, 136.73, 142.52, 154.95. Anal. calcd for C_20_H_21_ClN_2_O_2_S: C, 61.77; H, 5.44; N, 7.20; S, 8.24. Found: C, 61.51; H, 5.72; N, 7.41; S, 8.12.

##### 1-Cyclohexyl-2-(3,4-difluorophenyl)–5-(methylsulfonyl)–1*H*-benzo[*d*]imidazole (**24**)

Compound **24** was synthesized in line with the general method, starting from N^1^-cyclohexyl-4-(methylsulfonyl) benzene-1,2-diamine (0.93 mmol, 0.230 g) and sodium (3,4-difluorophenyl) (hydroxy)methanesulfonate (0.93 mmol, 0.229 g). The resulting product was purified by CC using an ethyl acetate/hexane (1:1) solvent system (0.224 g, 67.0% yield). M.P: 196.8 °C, MS (ESI^+^) [M + H]^+^ (%): 391.56 (100%). ^1^H NMR (400 MHz, DMSO-*d*_6_): δ ppm 1.23–1.42 (m, 3H, cyclohexyl), 1.64 (d, *J* = 11.2 Hz 1H, cyclohexyl), 1.83 (d, *J* = 12.8 Hz, 2H, cyclohexyl), 1.97 (d, *J* = 10.8 Hz, 2H, cyclohexyl), 2.21–2.30 (m, 2H, cyclohexyl), 3.24 (s, 3H, CH_3_), 4.21–4.23 (m, 1H, cyclohexyl), 7.53–7.57 (m, 1H), 7.67–7.74 (m, 1H), 7.78–7.83 (m, 2H), 8.17 (d, *J* = 8.8 Hz, 1H), 8.23 (d, *J* = 2 Hz, 1H). Anal. calcd for C_20_H_20_F_2_N_2_O_2_S: C, 61.52; H, 5.16; N, 7.17; S, 8.24. Found: C, 61.81; H, 5.54; N, 7.30; S, 8.18.

##### 2-(4-Chlorophenyl)–1-cyclohexyl-5-(ethylsulfonyl)–1*H*-benzo[*d*]imidazole (**25**)

Compound **25** was prepared based on the general method starting from *N*^1^-cyclohexyl-4-(ethylsulfonyl) benzene-1,2-diamine (0.96 mmol, 0.253 g) and sodium (4-chlorophenyl) (hydroxy)methanesulfonate (0.96 mmol, 0.236 g). The resulting product was purified by CC using an ethyl acetate/hexane (2:1) solvent system (0.167 g, 46.3% yield). M.P: 249.8 °C, MS (ESI^+^) [M + H]^+^ (%): 403.5 (100%). ^1^H NMR (400 MHz, DMSO-*d*_6_): δ ppm 1.10 (t, 3H), 1.29 (m, *J* = 7.6 Hz, 3H, cyclohexyl), 1.63 (d, *J* = 1.2 Hz, 1H, cyclohexyl), 1.82 (d, *J* = 1.2 Hz, 2H, cyclohexyl), 1.93 (d, *J* = 1.2 Hz, 2H, cyclohexyl), 2.21–2.30 (m, 2H, cyclohexyl), 3.29 (q, *J* = 8.0 Hz, 2H), 4.25 (m, 1H, cyclohexyl), 7.66–7.74 (m, 5H), 8.15–8.17 (m, 2H). ^13^C NMR (DMSO-*d*_6_): δ ppm 7.36, 24.29, 25.39, 30.45, 49.68, 57.12, 114.00, 119.94, 121.31, 128.81, 128.99, 131.33, 131.97, 135.15, 136.87, 142.59, 154.98. Anal. Calcd for C_21_H_23_ClN_2_O_2_S: C, 62.60; H, 5.75; N, 6.95; S, 7.96. Found: C, 62.77; H, 6.29; N, 7.15; S, 8.03.

##### 1-Cyclohexyl-2-(3,4-difluorophenyl)–5-(ethylsulfonyl)–1*H*-benzo[*d*]imidazole (**26**)

Compound **26** was prepared per the general method, beginning with *N*^1^-cyclohexyl-4-(ethylsulfonyl) benzene-1,2-diamine (0.86 mmol, 0.227 g) and sodium (3,4-difluorophenyl) (hydroxy) methanesulfonate (0.86 mmol, 0.212 g). The resulting product was purified by CC using an ethyl acetate/hexane (1:1) solvent system (0.119 g, 36.6% yield). M.P: 193 °C, MS (ESI^+^) [M + H]^+^ (%): 405.52 (100%). ^1^H NMR (400 MHz, DMSO-*d*_6_): δ ppm 1.09 (t, *J* = 7.6 Hz, 3H), 1.22–1.43 (m, 3H, cyclohexyl), 1.62 (d, *J* = 1.0 Hz, 1H, cyclohexyl), 1.82 (d, *J* = 1.2 Hz, 2H, cyclohexyl), 1.95 (d, *J* = 1.2 Hz, 2H, cyclohexyl), 2.19–2.28 (m, 2H, cyclohexyl), 3.33 (q, *J* = 12.4 Hz, 2H), 4.20–4.26 (m, 1H, cyclohexyl), 7.51–7.57 (m, 1H), 7.65–7.82 (m, 3H), 8.15–8.17 (m, 2H). Anal. calcd for C_21_H_22_F_2_N_2_O_2_S: C, 62.36; H, 5.48; N, 6.93; S, 7.93. Found: C, 62.61; H, 5.71; N, 7.08; S, 7.91.

##### 1-(3,4-Difluorobenzyl)–2-(3,5-difluorophenyl)–5-(methylsulfonyl)–1*H*-benzo[*d*]imidazole (**27**)

Compound **27** was synthesized based on the general method starting from *N*^1^–3,4-difluorobenzyl-4-(methylsulfonyl) benzene-1,2-diamine (0.83 mmol, 0.260 g) and sodium (3,5-difluorophenyl) (hydroxy)methanesulfonate (0.83 mmol, 0.205 g). The resulting product was purified by CC using an ethyl acetate/hexane (1:2) solvent system (0.115 g, 31.8% yield). M.P: 170.3 °C, MS (ESI^+^) [M + H]^+^ (%): 435.65 (100%). ^1^H NMR (400 MHz, DMSO-*d*_6_): δ ppm 3.24 (s, 3H, CH_3_), 5.47 (s, 2H, CH_2_), 6.72–6.74 (m, 1H), 7.06–7.11 (m, 1H), 7.28–7.35 (m, 2H), 7.49–7.55 (m, 1H), 7.72–7.86 (m, 3H), 8.28 (d, *J* = 1.6 Hz, 1H). Anal. calcd for C_21_H_14_F_4_N_2_O_2_S: C, 58.06; H, 3.25; N, 6.45; S, 7.38. Found: C, 58.29; H, 3.45; N, 6.62; S, 7.58.

##### 1-(3,4-Difluorobenzyl)–2-(2,5-difluorophenyl)–5-(methylsulfonyl)–1*H*-benzo[*d*]imidazole (**28**)

Compound **28** was obtained according to the general method beginning with *N*^1^–3,4-difluorobenzyl-4-(methylsulfonyl) benzene-1,2-diamine (0.90 mmol, 0.280 g) and sodium (2,5-difluorophenyl) (hydroxy)methanesulfonate (0.90 mmol, 0.205 g). The resulting product was purified by CC using an ethyl acetate/hexane (1:2) solvent system (0.05 g, 12.8% yield). M.P: 168.3 °C, MS (ESI^+^) [M + H]^+^ (%): 435.63 (100%). ^1^H NMR (400 MHz, DMSO-*d*_6_): δ ppm 3.25 (s, 3H, CH_3_), 5.51 (s, 2H, CH_2_), 6.75–6.77 (m, 1H), 7.08–7.13 (m, 1H), 7.28–7.35 (m, 1H), 7.50–7.56 (m, 2H), 7.61–7.66 (m, 1H), 7.82–7.88 (m, 2H), 8.31 (s, 1H). Anal. Calcd for C_21_H_14_F_4_N_2_O_2_S: C, 58.06; H, 3.25; N, 6.45; S, 7.38. Found: C, 57.79; H, 3.29; N, 6.58; S, 7.36.

##### *N*-(4-(1-ethyl-5-(methylsulfonyl)–1*H*-benzo[*d*]imidazol-2-yl)phenyl) (**29**)

Compound **29** was obtained following the general method, starting from *N*^1^-ethyl-(methylsulfonyl) benzene-1,2-diamine (1.42 mmol, 0.304 g) and sodium (4-acetamidophenyl) (hydroxy) methanesulfonate (1.42 mmol, 0.351 g). The resulting product was purified by CC using an ethyl acetate/isopropyl (5:1) solvent system (0.121g, 23.9% yield). M.P: 215.1 °C, MS (ESI^+^) [M + H]^+^ (%): 358.58 (100%). ^1^H NMR (500 MHz, DMSO-*d*_6_): δ ppm 1.35 (t, *J* = 7.1 Hz, 3H), 2.11 (s, 3H), 3.25 (s, 3H), 4.42 (q, *J* = 7.1 Hz, 2H), 7.80 (dd, J = 8.4 Hz, *J* = 2 Hz, 4H), 7.95 (d, *J* = 8.6 Hz, 1H), 8.23 (s, 1H), 10.26 (s, 1H). ^13^C NMR (DMSO-*d*_6_): δ ppm 14.95, 24.18, 44.25, 111.62, 118.57, 118.84, 120.76, 123.77, 129.86, 134.56, 138.55, 141.12, 142.00, 155.47, 168.83. Anal. calcd for C_18_H_19_N_3_O_3_S0.7H_2_O: C, 58.42; H, 5.55; N, 11.35; S, 8.66. Found: C, 58.26; H, 5.86; N, 11.20; S, 8.46.

##### 1-Ethyl-2-(4-fluorophenyl)–5-(methylsulfonyl)–1*H*-benzo[*d*]imidazole (**30**)

Compound **30** was synthesized in line with the general method, starting from *N*^1^-ethyl-4-(methylsulfonyl) benzene-1,2-diamine (1.38 mmol, 0.296 g) and sodium (4-fluorophenyl) (hydroxy)methanesulfonate (1.38 mmol, 0.289 g). The resulting product was purified by CC using an ethyl acetate/hexane (4:1) solvent system (0.048 g, 10.9% yield). M.P: 198.9 °C, MS (ESI^+^) [M + H]^+^ (%): 319.59 (100%). ^1^H NMR (400 MHz, DMSO-*d*_6_): δ ppm 1.31 (t, *J* = 7.6 Hz, 3H), 3.23 (s, 3H), 4.36 (q, *J* = 7.2 Hz, 2H), 7.43 (t, *J* = 9.2 Hz, 2H), 7.83–7.87 (m, 3H), 7.94 (d, *J* = 8.8 Hz, 1H), 8.22 (s, 1H). ^13^C NMR (DMSO-*d*_6_): δ ppm 14.89, 44.22, 111.81, 116.03 (d, *J* = 21.8 Hz), 118.78, 120.96, 126.10 (d, *J* = 3.2 Hz), 131.69, (d, *J* = 8.3 Hz), 134.72, 138.41, 141.87, 154.72, 163.19 (d, *J* = 246.6 Hz). Anal. calcd for C_16_H_15_FN_2_O_2_S-0.08C_6_H_14_: C, 60.85; H, 4.99; N, 8.61; S, 9.85. Found: C, 60.88; H, 5.28; N, 8.66; S, 9.68.

##### 1-Butyl-5-(ethylsulfonyl)–2-(4-fluorophenyl)-1*H*-benzo[*d*]imidazole (**31**)

Compound **31** was obtained according to the general method starting from *N*^1^-butyl-4-(ethylsulfonyl) benzene-1,2-diamine (0.14 mmol, 0.446 g) and sodium (4-fluorophenyl) (hydroxy)methanesulfonate (0.14 mmol, 0.298 g). The resulting product was purified by CC using an ethyl acetate/hexane (1:2) solvent system (0.0608 g, 9.7%, yield). M.P: 146.1 °C, MS (ESI^+^) [M + H]^+^ (%): 361.65 (100%). ^1^H NMR (500 MHz, DMSO-*d*_6_): δ ppm 0.87 (t, *J* = 7.4 Hz, 3H), 1.10–1.16 (m, 4H), 1.63–1.66 (m, 2H), 3.26 (t, *J* = 5.5 Hz, 3H), 4.54 (t, *J* = 7.35 Hz, 2H), 7.44–7.48 (m, 2H), 7.80 (dd, *J* = 8.5 Hz, *J* = 1.7 Hz, 1H), 7.86–7.89 (m, 2H), 7.95 (d, *J* = 8.5 Hz, 1H), 8.18 (d, *J* = 1.5 Hz, 1H). ^13^C NMR (DMSO-*d*_6_): δ ppm 7.85, 13.72, 19.62, 31.56, 44.64, 50.15, 112.42, 116.47 (d, *J* = 21.7 Hz), 120.08, 122.14, 126.74 (d, *J =* 3.0 Hz), 126.75, 132.21 (d, *J =* 8.7 Hz), 132.61, 139.29, 142.29, 155.45, 163.60 (d, *J =* 246.8 Hz). Anal. calcd for C_19_H_21_FN_2_O_2_S: C, 63.31; H, 5.87; N, 7.77; S, 8.90. Found: C, 63.45; H, 6.15; N, 7.86; S, 8.85.

##### *N*-(4-(1-Butyl-5-(ethylsulfonyl)-1*H*-benzo[*d*]imidazol-2-yl) phenyl) Acetamide (**32**)

Compound **32** was synthesized according to the general method starting from *N*^1^-butyl-4-(ethylsulfonyl) benzene-1,2-diamine (0.15 mmol, 0.473 g) and sodium (4-acetamidophenyl) (hydroxy)methanesulfonate (0.15 mmol, 0.267 g). The resulting product was purified by CC using an ethyl acetate/hexane (4:1) solvent system (0.035 g, 4.8% yield). M.P: 179.9 °C, MS (ESI^+^) [M + H]^+^ (%): 400.66 (100%). ^1^H NMR (500 MHz, DMSO-*d*_6_): δ ppm 0.77 (t, *J* = 7.3 Hz, 3H), 1.10–1.18 (m, 4H), 1.64–1.67 (m, 2H), 2.11 (s, 3H), 3.31 (t, *J* = 4.5 Hz, 3H), 4.39 (t, *J* = 7.3 Hz, 2H), 7.75–7.83 (m, 5H), 7.93 (d, *J* = 8.6 Hz, 1H), 8.17 (s, 1H), 10.25 (s, 1H). ^13^C NMR (DMSO-*d*_6_): δ ppm 7.85, 13.75, 19.65, 24.61, 31.57, 44.67, 50.17, 112.21, 119.24, 119.85, 121.91, 124.42, 130.33, 132.45, 139.42, 141.50, 142.42, 156.20, 169.25. Anal. calcd for C_21_H_25_N_3_O_3_S: C, 63.13; H, 6.31; N, 10.52; S, 8.03. Found: C, 63.01; H, 6.43; N, 10.65; S, 8.00.

##### Methyl 4-(1-(3,4-Difluorobenzyl)-5-(methylsulfonyl)-1*H*-benzo[*d*]imidazol-2-yl) Benzoate (**33**)

Compound **33** was obtained in line with the general method starting from *N*^1^–3,4-difluorobenzyl-4- (methylsulfonyl) benzene-1,2-diamine (0.13 mmol, 0.348 g) and sodium hydroxyl-(4-methoxycarbonyl)phenyl methanesulfonate (0.13 mmol, 0.299 g). The resulting product was purified by CC using an ethyl acetate/hexane (1:3) solvent system (0.068 g, 13.4% yield). M.P: 408.7 °C, MS (ESI^+^) [M + H]^+^ (%): 457.67 (100%). ^1^H NMR (400 MHz, DMSO-*d*_6_): δ ppm 3.25 (s, 3H), 3.84 (s, 3H), 5.65 (s, 2H), 6.76–6.79 (m, 1H), 7.21–7.26 (m, 1H), 7.33–7.40 (m, 1H), 7.70–7.73 (m, 1H), 7.82–7.87 (m, 2H), 8.02–8.05 (m, 1H), 8.11–8.14 (m, 1H), 8.22–8.23 (m, 1H), 8.30 (s, 1H). Anal. calcd for C_23_H_18_F_2_N_2_O_4_S-0.3CH_3_OH: C, 60.04; H, 4.15; N, 6.01; S, 6.88. Found: C, 60.52; H, 3.97; N, 6.14; S, 7.02.

##### 2-(4-Chlorophenyl)-1-propyl-5-(propylsulfonyl)-1*H*-benzo[*d*]imidazole (**34**)

Compound **34** was prepared based on the general method, starting from *N*^1^-propyl-4-(propylsulfonyl) benzene-1,2-diamine (0.15 mmol, 0.437 g) and sodium (4-chlorophenyl) (hydroxy) methanesulfonate (0.15 mmol, 0.373 g). The resulting product was purified by CC using an ethyl acetate/hexane (1:2) solvent system (0.022 g, 3.4% yield). M.P: 172.8 °C, MS (ESI^+^) [M + H]^+^ (%): 377.63 (100%). ^1^H NMR (500 MHz, DMSO-*d*_6_): δ ppm 0.74 (t, *J* = 7.4 Hz, 3H), 0.91 (t, *J* = 7.4 Hz, 3H), 1.54–1.59 (m, 2H), 1.67–1.71 (m, 2H), 3.30–3.33 (m, 2H), 4.34 (t, *J* = 7.4 Hz, 2H), 7.69 (d, *J* = 8.5 Hz, 2H), 7.8 (dd, *J* = 8.5 Hz, *J* = 1.7 Hz 1H), 7.84 (d, *J* = 8.5 Hz, 2H), 7.98 (d, *J* = 6.8 Hz, 1H), 8.18 (d, *J* = 1.1 Hz, 1H). Anal. calcd for C_19_H_21_ClN_2_O_2_S-0.15CH_3_OH: C, 60.25; H, 5.70; N, 7.33; S, 8.40. Found: C, 60.45; H, 5.67; N, 7.22; S, 8.49.

##### 2-(2,5-Difluorophenyl)-1-(4-fluorobenzyl)-5-(methylsulfonyl)-1*H*-benzo[*d*]imidazole (**35**)

Compound **35** was obtained following the general method starting from *N*^1^-(4-fluorobenzyl)-4-(methylsulfonyl) benzene-1,2-diamine (0.64 mmol, 0.199 g) and sodium (2,5-difluorophenyl) (hydroxy) methanesulfonate (0.64 mmol, 0.171 g). The resulting product was purified by CC using an ethyl acetate/hexane (1:10) solvent system (0.082 g, 29.1% yield). M.P: 236.1 °C, MS (ESI^+^) [M + H]^+^ (%): 417.43 (100%). ^1^H NMR (400 MHz, DMSO-*d*_6_): δ ppm 3.24 (s, 3H), 5.47 (s, 2H), 6.96–6.99 (m, 2H), 7.05–7.10 (m, 2H), 7.29–7.33 (m, 1H), 7.50–7.55 (m, 1H), 7.71–7.77 (m, 1H), 7.80–7.85 (m, 2H), 8.27 (s, 1H). Anal. calcd for C_21_H_15_F_3_N_2_O_2_S-0.1C_6_H_14_: C, 61.03; H, 3.88; N, 6.59; S, 7.54. Found: C, 60.67; H, 3.71; N, 6.74; S, 7.69.

##### *N*-(4-(1-(4-Fluorobenzyl)-5-(methylsulfonyl)-1*H*-benzo[*d*]imidazol-2-yl)phenyl) Acetamide (**36**)

Compound **36** was synthesized based on the general method starting from *N*^1^-(4-fluorobenzyl)-4-(methylsulfonyl) benzene-1,2-diamine (0.86 mmol, 0.272 g) and sodium (4-acetamidophenyl) (hydroxy)methanesulfonate (0.86 mmol, 0.180 g). The resulting product was purified by CC using an ethyl acetate/hexane (1:5) solvent system 0.080 g, 19.8% yield. M.P: 165.7 °C, MS (ESI^+^) [M + H]^+^ (%): 438.58 (100%). ^1^H NMR (500 MHz, DMSO-*d*_6_): δ ppm 2.09 (s, 3H), 3.24 (s, 3H), 5.67 (s, 2H), 7.03–7.14 (m, 4H), 7.70–7.81 (m, 6H), 8.25 (s, 1H), 10.24 (s, 1H). ^13^C NMR (DMSO-*d*_6_): 24.31, 44.62, 47.59, 112.35, 116.18 (d, *J* = 21.5 Hz), 119.15, 119.24, 121.56, 123.94, 128.81 (d, *J =* 8.3 Hz), 130.30, 133.08 (d, *J =* 2.7 Hz), 135.42, 139.46, 141.68, 142.48, 156.36, 161.92, (d, *J =* 242.3 Hz), 169.25. Anal. calcd for C_23_H_20_FN_3_O_3_S-0.75CH_3_OH: C, 60.66; H, 4.73; N, 8.57; S, 6.54. Found: C, 60.84; H, 5.30; N, 8.89; S, 6.56.

### 2.4 Cell Viability Assay

MCF-7 cells were grown in RPMI-1640 cell culture growth medium supplemented with 10% fetal bovine serum and stabilized with 1.5% l-glutamine and 0.1 mg/mL gentamicin (Sigma-Aldrich) at 37 °C in a 5% CO_2_ incubator. An xCELLigence Real-Time Quantitative Cell Analyzer (Agilent) was used to assess the potential *in vitro* cytotoxic activities of compounds **23**–**36** against MCF-7 cells. Real-time cell viability was examined in a 16-well plate integrated with a bottom-placed microelectronic cell sensor array. Confluent MCF-7 cells were seeded in a plate at a density of 1 × 10^4^ cells per well in 200 μL of complete medium. The cells were allowed to grow overnight to facilitate their adhesion to the wells and treated with three different concentrations of the compounds (5, 50, and 100 μM) for 72 h.^26^ Vincristine (1 μM) was used as positive control. The cytotoxic effects of the synthesized compounds were compared with that of vincristine. In this way, the most toxic derivatives were determined. The candidate compounds, **23** and **27**, were identified to have demonstrated a significant cytotoxic effect compared to vincristine. These compounds were subsequently selected for gene expression analysis and molecular docking studies. The degree of cytotoxicity was determined by measuring the IC_50_ values at 72 h. The IC_50_ values for compounds **23** and **27** were determined by using the RTCA Software 2.0 program of the xCELLigence Real-Time Cell Analyzer.

### 2.5 Gene Expression Analysis

The methods for gene expression analysis have been described in our previous work on indole-thiazolidinedione derivatives.^26^

#### 2.5.1 RNA Isolation

The High Pure RNA Isolation Kit (Roche Life Sciences, Germany) was used for the purification of total RNA from cultured cells. The IC_50_ doses of the two most cytotoxic derivatives-compounds **23** (4.7 μM) and **27** (10.9 μM) were administered to MCF-7 cells and incubated for 72 h. Vincristine (1 μM) and untreated MCF-7 cells were used as the positive and negative controls, respectively. RNA was isolated from cells using a High Pure RNA Isolation Kit (Roche Life Sciences, Germany) according to the manufacturer’s instructions. The concentration and purity of RNA samples were determined using an AlphaSpec (α Innotech) spectrophotometer. RNA samples with an optical density (OD_260_/OD_280_) ratio of 1.8–2.0, which denotes good purity, were selected based on their absorbance readings.

#### 2.5.2 cDNA Synthesis

RNA samples with a standardized concentration of 1000 ng for each group were used for cDNA (cDNA) synthesis. The cDNA was synthesized from total RNA using a random hexamer of Transcriptor High Fidelity cDNA Synthesis Kit (Roche Life Sciences, Germany) following the protocols provided by the manufacturer.

#### 2.5.3 Quantitative Real-Time Polymerase Chain Reaction (qRT-PCR)

qRT-PCR experiments (Real-time Ready Custom qPCR Assays, Roche Life Sciences) were performed on 96-well plates using the Roche Light Cycler480 instrument. Primers were selected for the 45 genes studied and the arrays were designed to permit duplicate experiments. To normalize the gene expression analyses, housekeeping genes (GAPDH, G6PD, and ACTB) in the array were employed as highlighted in Table 1.

**Table 1:**
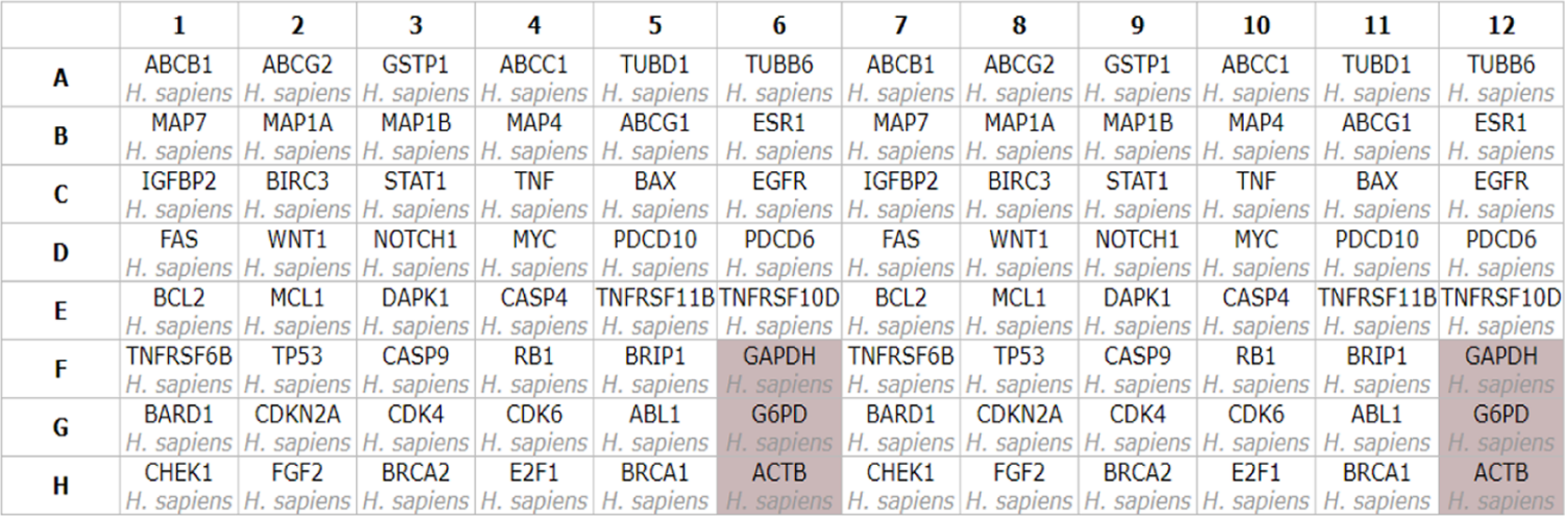
PCR Array Design and Genes in the Array^a^

a Reproduced with permission from ref (26). Copyright 2021 Centre National de la Recherche Scientifique (CNRS) and the Royal Society of Chemistry.

### 2.6 Statistical Analysis

To determine the relative changes in gene expression, qRT-PCR was performed in duplicate for each group, and the Ct values were calculated (−2^ΔΔCt^) for each gene. The ΔΔCt value was calculated by comparing the Ct value of the gene of interest to that of a control gene in both treated and untreated MCF-7 cells. Specifically, the ΔΔCt value was determined by subtracting the Ct value of the gene of interest in the treated cell line from the Ct value of the same gene in the untreated cell line and then subtracting the average Ct value of the housekeeping genes in the treated cell line from the average Ct value of the housekeeping genes in the untreated cell line. The fold change in gene expression was calculated using the ΔΔCt method and analyzed using the SPSS 16.0 program. Results were considered statistically significant if the difference was *p* < 0.05 or if the gene expression changed by 2-fold.

### 2.7 Computational Studies

#### 2.7.1 Molecular Docking

The crystal structure of Bcl-2 (PDB ID: 6O0K, resolution:1.62 Å), the most prominent gene in the gene expression analysis, was obtained from the RCSB Protein Data Bank.^27^ The crystal structures were imported into AutoDockTools 1.5.6.^28^ All water molecules were deleted, and the grid box parameters based on the bound co-ligands were defined as follows: Size *X* = 40, Size *Y* = 40, Size *Z* = 56, Center *X* = −13.088, Center *Y* = 0.0, Center *Z* = −10.15, spacing = 0.375. Polar hydrogen and Gasteiger charges were also added. The two-dimensional (2D) chemical structures of compounds **23** and **27** were sketched using ChemDraw Ultra Version 12.0 software. Each molecule was minimized using Merck Molecular (MMFF94) and Universal Force Fields (UFF), with the steepest descent algorithm and a convergence value of 10^–7^. The ligands were transformed into PDB format using Avogadro software.^29^ Subsequently, Gasteiger charges and torsion were incorporated into the ligand files using AutoDockTools. The prepared ligands were docked against the protein target using AutoDock Vina.^30^ Interaction diagrams were created, and docking results were visualized using the PyMOL Molecular Graphics System v2.5.4.^31^ Vincristine was used as the reference ligand since it was the positive control in the cytotoxic activity assay.

##### 2.7.1.1 Docking Validation

Validation of the docking pipeline was done using the crystallographic PDB file of Bcl-2 (6O0K). LBM entry (venetoclax) in the protein file was downloaded as model SDF and converted into PDBQT format using AutoDockTools software. This ligand file was then re-docked using the grid parameters obtained from the binding site of LBM. Finally, the re-docked output and the co-ligand LBM files were converted into MOL2 format using OpenBabel GUI. These files were then used as inputs for DockRMSD software^32^ to obtain the RMSD value.

#### 2.7.2 Molecular Dynamics Simulation

The molecular dynamics (MD) simulations were executed using GROMACS version 20212, which is a state-of-the-art software application tailored for simulating proteins, lipids, and nucleic acids with a high degree of accuracy and efficiency.^33^ The initial system setup, including solvation and ion placement, was performed using the CHARMM-GUI web-based interface,^34^ which is renowned for its comprehensive and user-friendly tools for generating ready-to-simulate input files with Charmm36m force fields.^35^ Before the main simulation run, the system was equilibrated through a protocol involving a 0.25 ns pre-equilibration under constant volume (NVT) and constant pressure (NPT) ensembles to achieve a stable environment at 303.15 K and 1 atm, respectively. This process allowed the system to reach thermal and pressure equilibrium, ensuring a realistic biological context for the simulations. The MD simulation itself extended over a substantial 200 ns, providing ample temporal scope to observe the dynamic behavior and interactions within the biomolecular system. Through a rigorous sequence of energy minimization, and NVT/NPT equilibrations, followed by the production run, the system’s energy components, structural integrity, and temporal interactions were monitored and analyzed. Postsimulation analyses, including root-mean-square deviation (RMSD), and hydrogen bond interactions, were employed to elucidate the stability and conformational changes of the protein–ligand complexes. The end point of these analyses was the MM/PBSA calculation with gmx_MMPBSA tools,^36^ conducted on a select 500-frame window between 150 and 200 ns, to derive the free energy estimates indicative of the binding affinities of the investigated compounds. The analysis is anchored by the following equation:
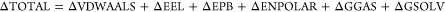


#### 2.7.3 Density Functional Theory (DFT) Method

All theoretical calculations were made using ORCA^37^ and Avogadro^38^ programs. Initially, the structures were drawn and executed in Avogadro wherein the input files for the ORCA program were created and parameters set as follows: single-point energy for calculation, DFT for method, and *def2-TZVPP* for basis. In the control section, the method was set as DFT. DFT Functional was set to *B3LYP*. Print options were set to MOs and Basis sets. Then, the input was generated and used in ORCA software. Molecular orbitals, MEP maps, and their energy values were visualized using Avogadro GUI. The energy and dipole moment values were calculated using ORCA software.

### 2.8 Prediction of ADME Properties

The SwissADME online program (https://www. swissadme.ch) was used to predict the ADME properties of the synthesized compounds.^39^ The 2D chemical structures of the compounds were imported into the program and converted to canonical simplified molecular input line entry system (SMILES) strings. The SMILES string for the standard drug vincristine was obtained from PubChem database. Molecular parameters including log Po/*w*, blood-brain barrier (BBB) permeation, *P*-glycoprotein substrate characteristics, cytochrome-P450 enzyme inhibition, and suitability to the Lipinski and Veber filters were calculated.^40,41^

## 3 Results and Discussion

### 3.1 Chemistry

**Scheme 1:**
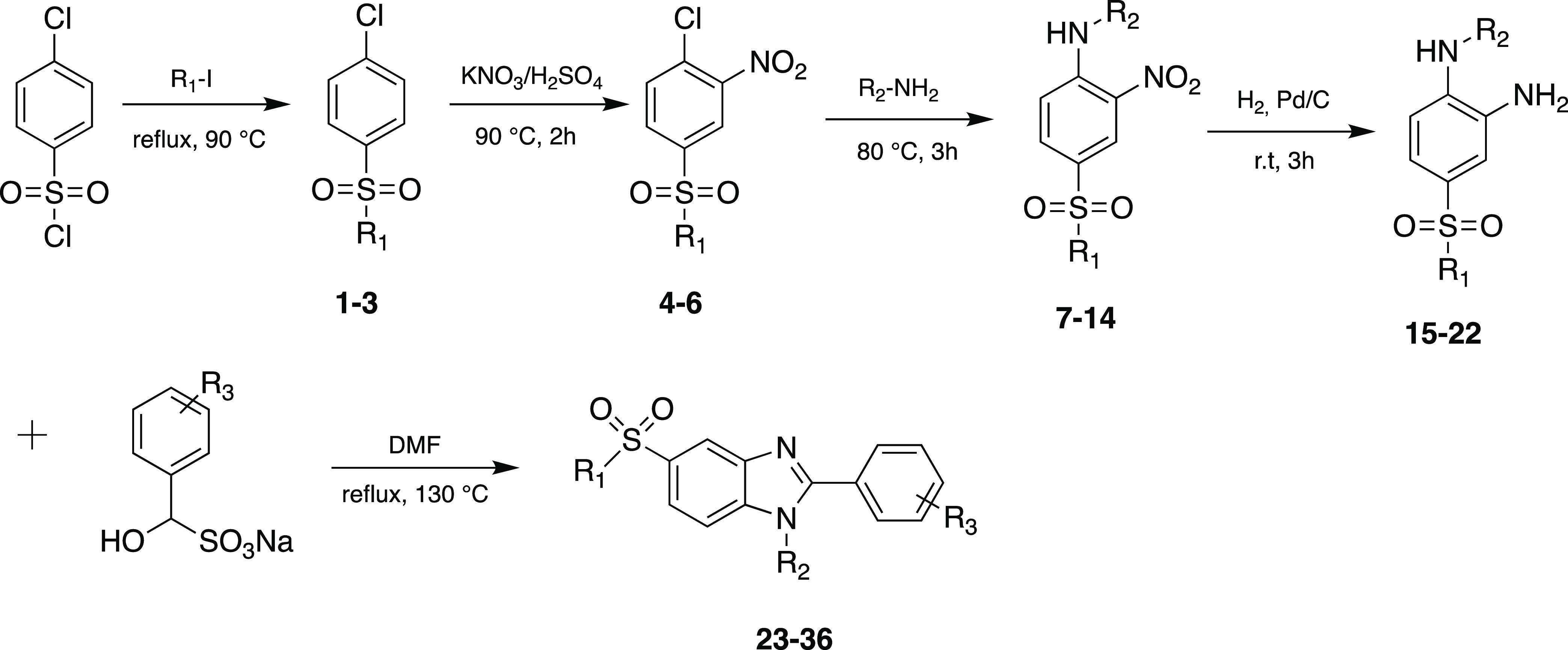
Synthesis of Alkylsulfonyl 1*H*-Benzo[*d*]imidazole Compounds **23**–**36**

The benzimidazole scaffold is a versatile pharmacophore of choice for the design of various therapeutic agents in medicinal chemistry.^42^ In this study, a series of new substituted benzimidazole derivatives (**23**–**36**) were successfully synthesized in continuation of our previous work on the benzimidazole scaffold. The synthetic route is simple as shown in Scheme 1. Under controlled conditions, the syntheses were achieved, and reactions were monitored stepwise with silica gel thin-layer chromatography until completion. Compounds were purified by crystallization and silica gel column chromatography. The structures of the synthesized compounds were confirmed by ^1^H NMR, ^13^C NMR, electrospray ionization mass spectrometry (ESI-MS), and elemental analysis. The synthesized compounds bear different alkylsulfonyl groups at the benzene end of the structure as shown in Table 2. The alkyl (R_1_) member series includes methyl, ethyl, and propyl. The R_3_ moiety is predominantly characterized by halogen substituents, specifically chloro- and fluoro-, followed by acetamido and methoxycarbonyl groups. Alkylsulfonyl substituent is present on the benzene ring at the fifth position of the benzimidazole ring. One of the two nitrogen atoms on the imidazole ring bears the R_2_ group which contains several substituents such as cyclohexyl and disubstituted benzenes. The corresponding spectra for the synthesized compounds analyzed by ^1^H NMR, ^13^C NMR, and ESI-MS are given in the Supporting Information, while the physicochemical and elemental analyses data are provided in Section 2.

**Table 2:**
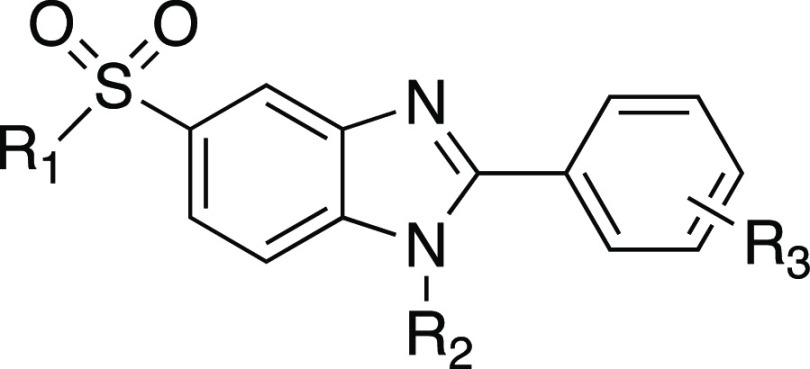
Structure of Alkylsulfonyl 1*H*-Benzo[*d*]imidazole Derivatives **23**-**36** and Their Effective Dose Range against MCF-7 Breast Cancer Cells^a^

				dose (μM)			
comp.	R_1_	R_2_	R_3_	10	100	tox. range	selected compounds*, **
**23**	–CH_3_	cyclohexyl	4-chloro	toxic	toxic	1–10*	most toxic*
**24**	–CH_3_	cyclohexyl	3,4-difluoro	toxic	toxic	10–60	
**25**	–C_2_H_5_	cyclohexyl	4-chloro	toxic	toxic	10–60	
**26**	–C_2_H_5_	cyclohexyl	3,4-difluoro	nontoxic	toxic	20–80	
**27**	–CH_3_	3,5-difluorobenzyl	3,4-difluoro	toxic	toxic	5–40*	second most toxic**
**28**	–CH_3_	3,4-difluorobenzyl	2,5-difluoro	toxic	toxic	5–40	
**29**	–CH_3_	ethyl	4-NHCOCH_3_	nontoxic	toxic	20–80	
**30**	–CH_3_	ethyl	4-fluoro	toxic	toxic	5–40	
**31**	–C_2_H_5_	butyl	4-fluoro	toxic	toxic	5–40	
**32**	–C_2_H_5_	butyl	4-acetamido	nontoxic	toxic	20–80	
**33**	–CH_3_	3,4-difluorobenzyl	4-COOCH_3_	nontoxic	toxic	20–80	
**34**	–C_3_H_7_	propyl	4-chloro	nontoxic	toxic	20–80	
**35**	–CH_3_	4-fluorobenzyl	2,5-difluoro	toxic	toxic	5–40	
**36**	–CH_3_	4-fluorobenzyl	4-NHCOCH_3_	toxic	toxic	1–50	
VCR				toxic	toxic	1	positive control

a Comp.; compounds, VCR; vincristine, Tox.; toxicity.

### 3.2 Evaluation of Cytotoxic Activity

Breast cancer is the predominant malignancy in terms of incidence and mortality in women worldwide. It has even eclipsed lung cancer as the most frequently diagnosed cancer with an estimated 2.3 million new cases in 2020 alone.^1^ Hence, there is a compelling need to develop newer compounds with remarkable anticancer properties to mitigate this health challenge. In this study, we investigated the anticancer activity of the newly synthesized alkylsulfonyl 1*H*-benzo[*d*]imidazole derivatives, and the results are shown in Table 3 which indicates most of the compounds are cytotoxic against the MCF-7 cells. Vincristine was used as a reference drug and based on its IC_50_ value, low dose and high dose values were selected for the 14 synthesized benzimidazole derivatives. At a dose of 10 μM, compounds **26**, **29**, **32**, **33**, and **34** were nontoxic to MCF-7 cells, while other remaining compounds showed toxicity. Two compounds, **23** (R_1_ = methyl, R_2_ = cyclohexyl, R_3_ = 4-chloro) and **27** (R_1_ = methyl, R_2_ = 3,5-difluorobenzyl, R_3_ = 3,4-difluoro), showed the greatest cytotoxic activity with dose ranges of 1–10 and 5–40 μM, respectively. Hence, the MCF-7 cells were less viable in the presence of the synthesized compounds **23** (4.7 μM) and **27** (10.9 μM) which were the most potent derivatives. Consequently, these two most potent derivatives were selected and subjected to gene expression and computational studies. Despite the relative margin in IC_50_ values, the *in vitro* cytotoxic effect of these two compounds closely compares to that of standard drug vincristine. Several benzimidazole derivatives displayed good activity against diverse cancer cell lines including lung cancer, non-small-cell lung cancer, leukemia, melanoma, and breast cancer.^43,44^ Singh and Tandon^44^ reported 2-aryl-substituted 2-bis-1*H*-benzimidazoles showed potent growth inhibitory effect against MCF-7 with IC_50_ within the range 1.3–16.8 μM. Similarly, several novel fluorinated benzimidazole-based compounds (*N*-fluoroaryl benzimidazole derivatives) displayed *in vitro* cytotoxicity against MCF-7 at low micromolar doses.^45^ Several studies have explored the potential anticancer properties of benzimidazoles. To improve the efficacy of cancer treatment, researchers worldwide continue to explore the synthesis of novel benzimidazole compounds that show improved selectivity about their targets, reducing nonspecific toxicities and adverse reactions.^46^ The benzimidazole scaffold continues to garner increasing interest in medicinal chemistry due to its remarkable anticancer activity and this has led to the approval of several benzimidazole-based compounds as commercial anticancer agents in the past decade by the United States Food and Drug Administration.^47^

**Table 3:** Fold Changes in Gene Expression Levels in MCF-7 Cells after Treatment with Compounds **23** and **27**

	control	vincristine		compound **23**		compound **27**	
gene	Ct	Ct	fold change −2^ΔΔCt^	Ct	fold change −2^ΔΔCt^	Ct	fold change −2^ΔΔCt^
ABCC1	31.00	30.00	+4	31.00	+2	29.00	+4
TUBD1	31.00	34.00	–2	31.00	+2	31.00	
MAP7	25.00	30.00	–16	27.00	–2	29.00	–16
MAP4	28.00	33.00	–16	31.00	–4	31.00	–8
ABCG1	34.00	33.00	+4	34.00	+2	31.00	+8
ESR1	30.00	33.00	–4	32.00	–2	31.00	–2
BIRC3	34.00	35.00		35.00		35.00	–2
STAT1	30.00	30.00	+2	35.00	–16	34.00	–16
MYC	33.00	35.00	–2	35.00	–2	35.00	–4
PDCD10	33.00	35.00	–2	33.00	+2	33.00	
BCL-2	27.00	31.00	–8	35.00	–128	35.00	–256
MCL-1	32.00	35.00	–4	30.00	+8	35.00	–8
TNFRSF6B	34.00	35.00		35.00		35.00	–2
TP53	27.00	28.00		27.00	+2	24.00	+8
RB	30.00	30.00	+2	35.00	–16	33.00	–8
CDK4	32.00	35.00	–4	35.00	–4	34.00	–4
ABL1	29.00	30.00		33.00	–8	32.00	–8
BRCA1	33.00	34.00		33.00	+2	40.00	–128
FAS	35.00	32.00	+16	35.00	+2	35.00	
CASP9	35.00	34.00	+4	35.00	+2	32.00	+8
BRIP1	35.00	33.00	+8	32.00	+16	35.00	
CDK6	35.00	34.00	+4	36.00		32.00	+8
MAP1B	35.00	36.00		33.00	+8	35.00	
PDCD6	35.00	36.00		36.00		33.00	+4

### 3.3 Gene Expression Analysis

In the gene expression analysis, we selected 45 genes related to microtubule organization, tumor suppression, apoptosis, cell cycle, and proliferation as shown in Table 1. Vincristine, a prominent tubulin polymerase inhibitor was used as positive control. Results showed that gene expression changes in MCF-7 for compounds **23** and **27** were comparably closer to vincristine. Furthermore, the gene expression levels of certain genes were more significantly altered by compounds **23** and **27** than by vincristine (Table 3). The prominent mechanisms of anticancer agents involve inhibition of DNA synthesis, DNA intercalation, microtubule inhibition, transcription regulation, enzyme inhibition, etc. Targeted therapy has emerged as one of the novel strategies in cancer chemotherapy, and most drugs exert their action by targeting protein kinases, tyrosine kinases, phosphoinositide-3 kinase, and structural proteins.^47^ The molecular mechanism of action of benzimidazoles also involves binding to tubulin proteins,^48^ thereby disrupting microtubular cell structure and preventing vital cellular functions. Among the studied microtubule-associated genes, MAP4 and MAP7 but not TUBD1 gene expressions were found to be markedly downregulated in compound **23**- and compound **27**-treated MCF-7 cells showing their inhibitory effects on the microtubule regulation. Furthermore, among the genes related to cell proliferation, ESR1, MYC, and CDK4 expression levels were found to be downregulated as in vincristine, compounds **23**- and **27**-treated cells as shown in Table 3. However, ABL-1 gene expression was only downregulated in compound **23**- and compound **27**-treated MCF-7 cells but not in the vincristine-treated group. Being a protooncogene, ABL-1 is involved in several fundamental cellular processes including cell division, adhesion, and differentiation. Our result indicates that the synthesized benzimidazole derivatives have more inhibitory effects on ABL-1 gene expression as compared to vincristine. BIRC3, BCL-2 and MCL-1 are antiapoptotic genes and their overall expression levels were markedly downregulated in MCF-7 cells after treatment with compound **27**. The BCL-2 gene had the most significant downregulation, with a fold change of 256 provoked by treatment with compound **27**. The expression of the BCL-2 gene was also significantly decreased by a 123-fold change after treatment with compound **23**. However, a similar significant impact on the expressions of BIRC3 and MCL-1 genes was not detected. There were no fold changes in BIRC3 expression following treatment with vincristine and compound **23**, except in compound **27** with a very low fold change of 2. Vincristine and compound **27** produced downregulation of MCL-1 gene expression with fold changes of 4 and 8, respectively. These results indicate that compound **27** has the highest effect in terms of inhibiting antiapoptotic gene expressions in comparison with compound **23** and vincristine. BCL-2 is an antiapoptotic gene and is usually overexpressed in breast cancer cells.^49^ However, our compounds suppressed the overexpression of this protein which indicates they could be potent inhibitors of the protein. Ilhan et al.^50^ analyzed BCL-2 gene and protein expression levels by qRT-PCR and Western blot analysis, respectively. They reported novel benzimidazole derivatives induced apoptosis and displayed BCL-2 inhibitory activity on a panel of cancer cell lines consisting of T98G glioblastoma, PC3 prostate, MCF-7 breast, and H69AR lung cancer. In tumor suppressor and apoptotic genes, we observed salient differences in gene expression levels in drug-treated and untreated groups. Marked overexpression of p53 was observed in compound **23**- and compound **27**-treated MCF-7 cells but not in vincristine-treated groups. Fas was highly overexpressed in the vincristine-treated group, but the effect is lower compared to cells treated with synthesized compounds. However, both BRCA1 and BRIP1 overexpression were only observed in the compound **23**-treated group. This finding is valuable because BRCA1 and BRIP1 are essential tumor suppressor genes and their proper functioning in the cell is necessary for DNA damage response. The BRCA1 gene plays a crucial role in the repair of damaged DNA, or in destroying cells if the DNA cannot be repaired. Moreso, BRIP1 is a member of the RecQ DEAH helicase family and interacts with the BRTC repeats of breast cancer. Therefore, the proper functioning of these two genes is crucial for normal cellular functions. The results of our study showed that compound **23** may exert its cytotoxic effect through BRCA1/BRIP1. Within the context of these findings, we infer that our compounds are multitarget gene inhibitors because they exert cytotoxic activity by downregulating several genes. Both compounds **23** and **27** act as potent inhibitors of the BCL-2 gene, while compound **27** showed remarkable inhibitory activity against BRCA1 genes in MCF-7 breast cancer cells.

### 3.4 Molecular Docking

**Figure 2:**
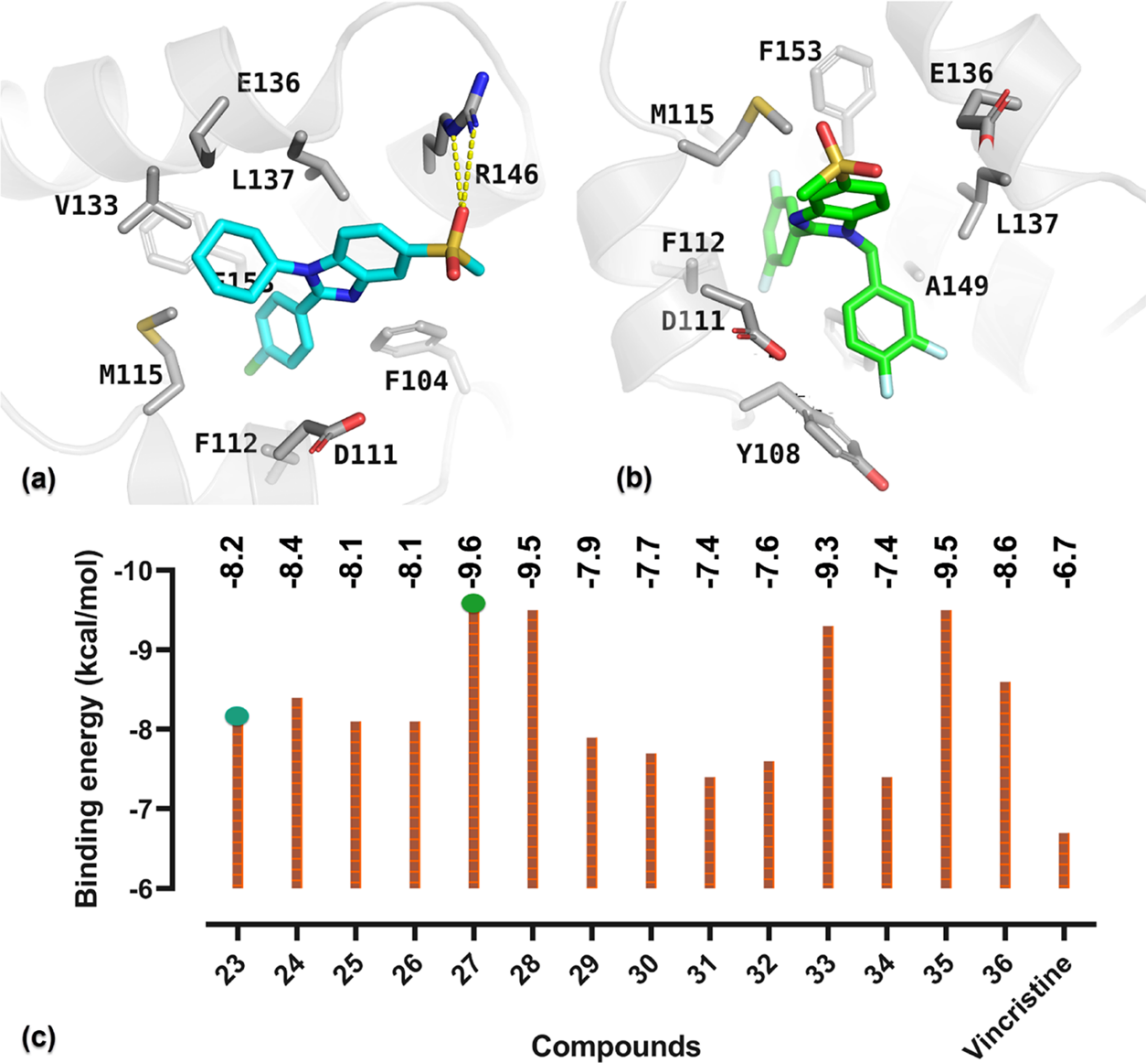
Molecular docking interactions and binding energies from AutoDock Vina. (a) Compound **23** docked in the Bcl-2 active site, detailing hydrogen bonds and hydrophobic interactions. (b) Compound **27**’s docking pose and specific interactions. (c) Comparison of the binding energies of various synthesized compounds obtained from AutoDock Vina simulations, assessing their binding affinities with vincristine serving as a benchmark compound.

The docking analysis pipeline was done according to the tested genes that are related to cellular events such as microtubule organization, tumor suppression, apoptosis, cell cycle, and proliferation. Among the 45 genes, the most important alterations were apparent for MAP4, MAP7, ABL-1, BCL-2, and BRCA1/BRIP1. Consequently, preliminary docking was made for the proteins that are related to these genes. However, only the analysis with BCL-2 yielded fruitful results. This enzyme is an antiapoptotic member of a protein family that is involved in the regulation of apoptotic cell death.^9^ Prevention of apoptosis occurs either by the separation of caspase proforms or by the prevention of the cytochrome c and apoptosis-inducing factor (AIF) release into the cytoplasm. After being released into the cytoplasm, cytochrome c and AIF activate caspases that cleave certain proteins, eventually causing apoptosis. Inhibition of this enzyme would, therefore, prevent the antiapoptotic effect, leading to apoptosis.^51^ Due to this effect of Bcl-2, we utilized it as a valid target for the apoptotic effect of our compounds. Grid box was defined as the venetoclax’s binding region in the Bcl-2 crystal structure (PDB ID: 6O0K).^52^ The synthesized compounds bound to this region and gave interactions similar to the reference ligand. For instance, compound **23**’s phenyl moiety created hydrophobic interactions with D111, F112 and the cyclohexyl ring interacted in the same way as with V133, E136, L137, and E156. Sulfonyl group of this compound offered H-bond interactions with R146 (Figure 2a). For compound **27**, many hydrophobic interactions with the amino acids Y108, D111, F112, M115, E136, L137, A149, and F153 are evident (Figure 2b). The docking score prediction by Vina showed the binding affinities of derivatives **23**–**36** fall between the range of −7.4 to −9.6 kcal/mol. Compound **27** had the best score of −9.6 kcal/mol followed by compounds **28** and **35** in second place, with a score of −9.5 kcal/mol. In third place, compound **33** offered an energy value of −9.3 kcal/mol. Similarly, the binding energy values for the other synthesized compounds are higher compared to the standard drug vincristine, with a score of −6.7 kcal/mol. Additionally, Prime/Molecular Mechanics Generalized Born Surface Area (Prime/MMGBSA)^53^ was calculated to predict the docking interaction energy of active compounds **23** and **27**. This was found to be −48.57 and −44.16 kcal/mol, respectively. In contrast to the docking interaction energies, compound **23** generated better interaction energy than **27**. By implication, this suggests that all our compounds showed higher binding affinity toward Bcl-2 protein than the standard drug. To assess the versatility of the docking process, an initial validation was done. First, Bcl-2 co-ligand venetoclax was separated from the 6O0K pdb file and then re-docked using the same grid parameters that would be used for the pipeline. The docking energy for the docking process was found to be good (−12.1 kcal/mol). Lastly, the results were evaluated in a three-dimensional environment and the root-mean-square deviation (RMSD) value between the two poses was calculated with DockRMSD online software.^32^ The conformation of the re-docked pose was significantly similar to the crystallographic pose (Supporting Figure 1), and the RMSD value was 0.574 Å, which is below 1 Å. This suggests that our docking process was valid and robust in investigating the process.

### 3.5 Molecular Dynamics Simulations

**Figure 3:**
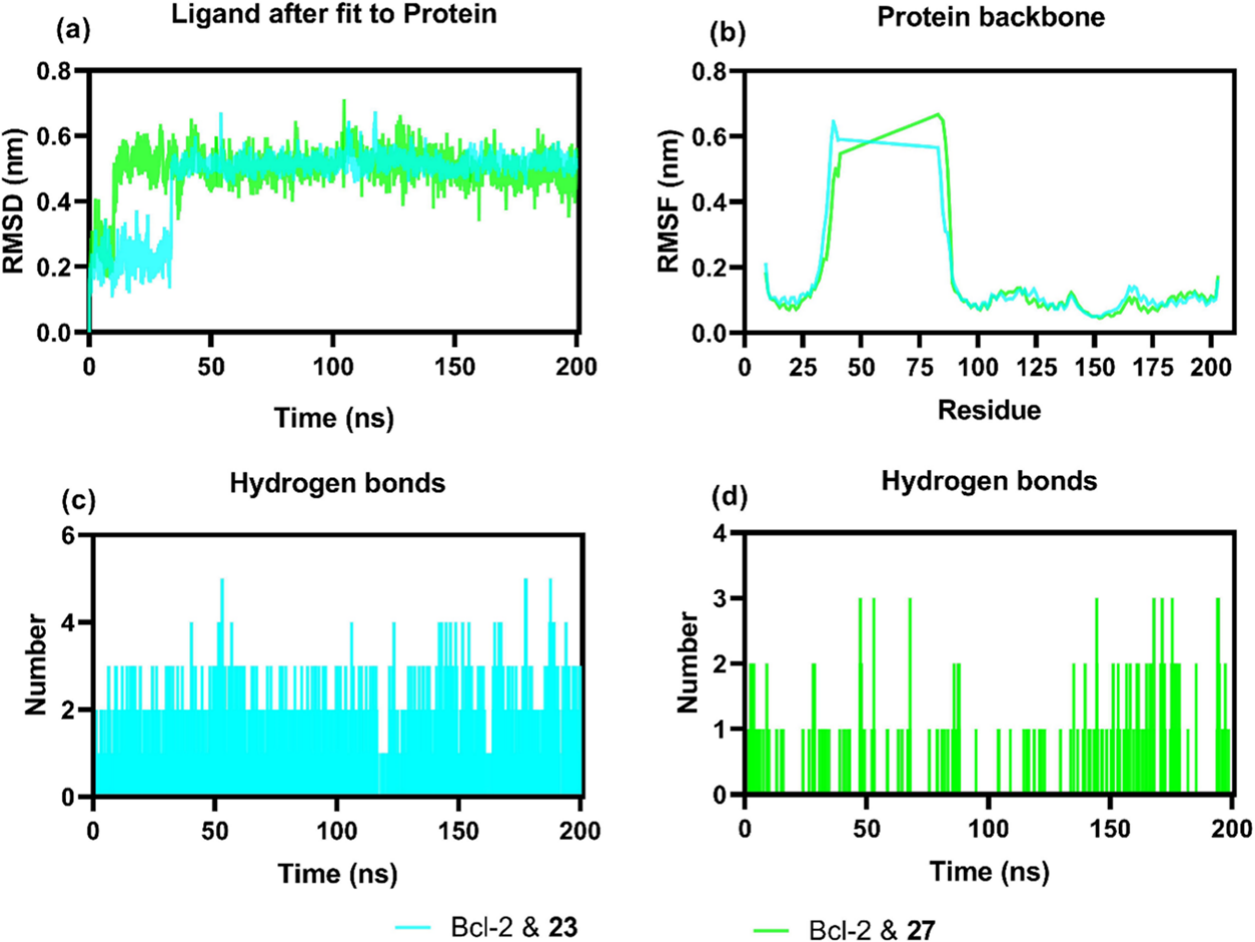
Molecular dynamics simulation trajectory analysis of Bcl-2 with **23** and **27**. (a) RMSD plot showing the stability of compounds **23** and **27** in the Bcl-2 active site, (b) RMSF plot expressing the flexibility of each residue in the Bcl-2 structure, (c, d) plots of the number of H bonds formed by compounds **23** and **27** in the Bcl-2 active site for 200 ns.

**Figure 4:**
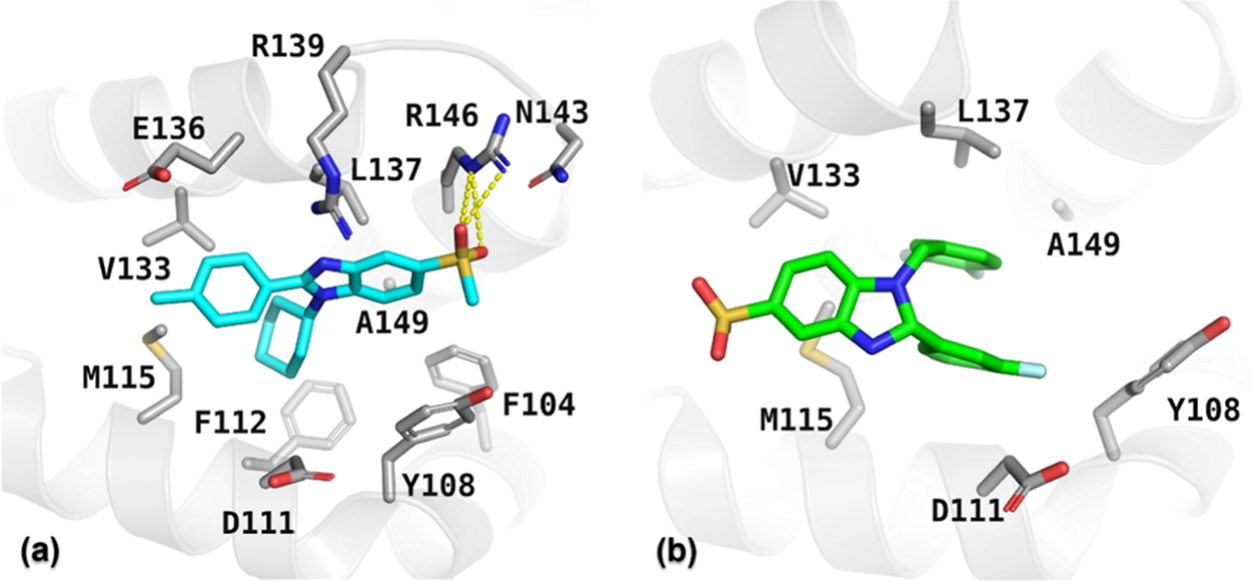
Binding modalities and protein–ligand interactions of compounds (a) **23** and (b) **27** in the Bcl-2 active pocket after 200 ns.

The stability of compounds **23** and **27** within the active site of Bcl-2 was visualized through the RMSD profiles depicted in the molecular dynamics simulations.^54^ As elucidated by the RMSD trajectory in Figure 3a, compounds **23** and **27** showed an initial conformational settling evidenced by an increase in RMSD values within the first 10 ns. This period of adjustment was promptly succeeded by a plateauing of the RMSD, indicating a stabilization of the ligand and Bcl-2 complexes, which was sustained for the remainder of the 200 ns simulation. The profiles remained predominantly below the 0.6 nm mark; a threshold often associated with stable ligand–protein interactions. In the context of protein flexibility, Figure 3b illustrates the root-mean-square fluctuation (RMSF) for the Bcl-2 backbone.^55^ This plot is instrumental in deciphering the flexibility across the protein sequence upon ligand binding. Certain residues are highlighted by pronounced peaks in the RMSF values, suggesting regions of increased mobility which may be critical for the protein’s functional dynamics or for accommodating the ligand. The hydrogen bond interactions, which are fundamental for the specificity and affinity of ligand binding, are quantitatively presented in Figure 3c,d. These graphs chronicle the fluctuations in the number of hydrogen bonds that compounds **23** and **27** form with Bcl-2. Compound **23** consistently forms a greater number of hydrogen bonds throughout the simulation, hinting at a possibly stronger and more stable interaction with the active site. In contrast, compound **27** exhibits fewer hydrogen bonds, indicating a more transient interaction with Bcl-2. The overall findings from the molecular dynamics simulations, encapsulated by the RMSD, RMSF, and hydrogen bond count plots, provide a multifaceted perspective on the interaction dynamics between Bcl-2 and the investigated ligands. The data from Figure 4 highlight the importance of considering both the stability and dynamics of ligand–protein interactions in the design and optimization of potential inhibitors. Understanding these molecular interactions at a detailed level is essential for the development of therapeutic strategies that target the Bcl-2 protein with high specificity and efficacy.

Complementing the dynamic simulation data,^56^Figure 4 presents the static snapshots of the final binding poses of compounds **23** and **27** within the Bcl-2 active site after 200 ns. Figure 4a captures the intricate network of interactions orchestrated by compound **23**, showcasing a dense array of hydrogen bonds and favorable hydrophobic contacts with key active site residues. This complex interaction landscape is critical for high-affinity binding and is reflected in the sustained occupancy and interaction pattern observed in the dynamic simulation.

Conversely, Figure 4b, depicting compound **27**, illustrates a sparser interaction profile with fewer hydrogen bonds and altered hydrophobic interactions. This less intricate binding mode correlates with the less favorable docking and binding free energy scores, and it is reflected in the higher RMSD values observed, indicating a less stable binding interaction.

**Figure 5:**
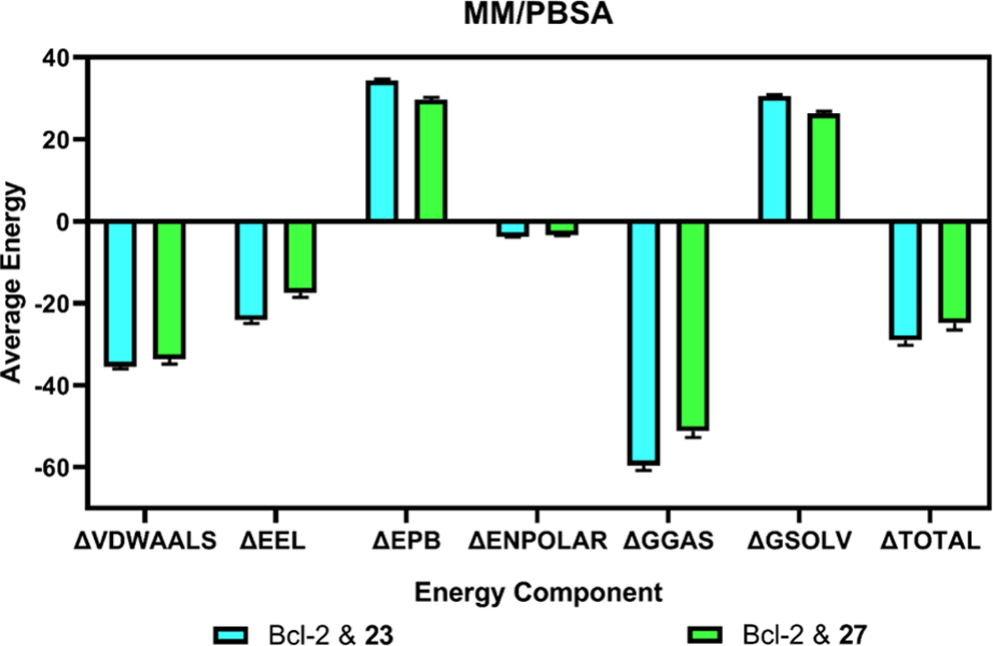
Energetic comparison of MM/PBSA calculations for **23** and **27** with Bcl-2, showing average component energies and the total binding free energies (ΔTOTAL) derived from MD simulations between 150 and 200 ns.

The Molecular Mechanics/Poisson–Boltzmann Surface Area (MM/PBSA) method represents a vital computational approach that allows for the quantification of the free energy of binding between a ligand and its target protein, an essential aspect in the elucidation of molecular interactions and the driving forces behind biological functions.^57^ Insights into the energetics of molecular interactions are critically illuminated through MM/PBSA calculations, as depicted in Figure 5, where the influence of van der Waals forces, electrostatic interactions, and solvation on binding affinity is highlighted. For compounds **23** and **27**, the ΔTOTAL—quantifying the overall free energy difference with values of −29.02 ± 1.20 and −24.82 ± 1.67 kcal/mol, respectively—emerges from a comprehensive assessment of individual energy contributions, including the molecular mechanics energies van der Waals and electrostatic interactions (ΔVDWAALS and ΔEEL), polar and nonpolar solvation energies (ΔEPB and ΔENPOLAR), along with the gas-phase free energy (ΔGGAS), and the solvation free energy (ΔGSOLV). The more pronounced negative ΔTOTAL for compound **23** suggests a stronger binding potential to Bcl-2, which may be pivotal for its inhibitory capacity, thereby offering valuable implications for the development of effective therapeutic inhibitors.

### 3.6 Molecular Reactivity and Stability Analysis

**Figure 6:**
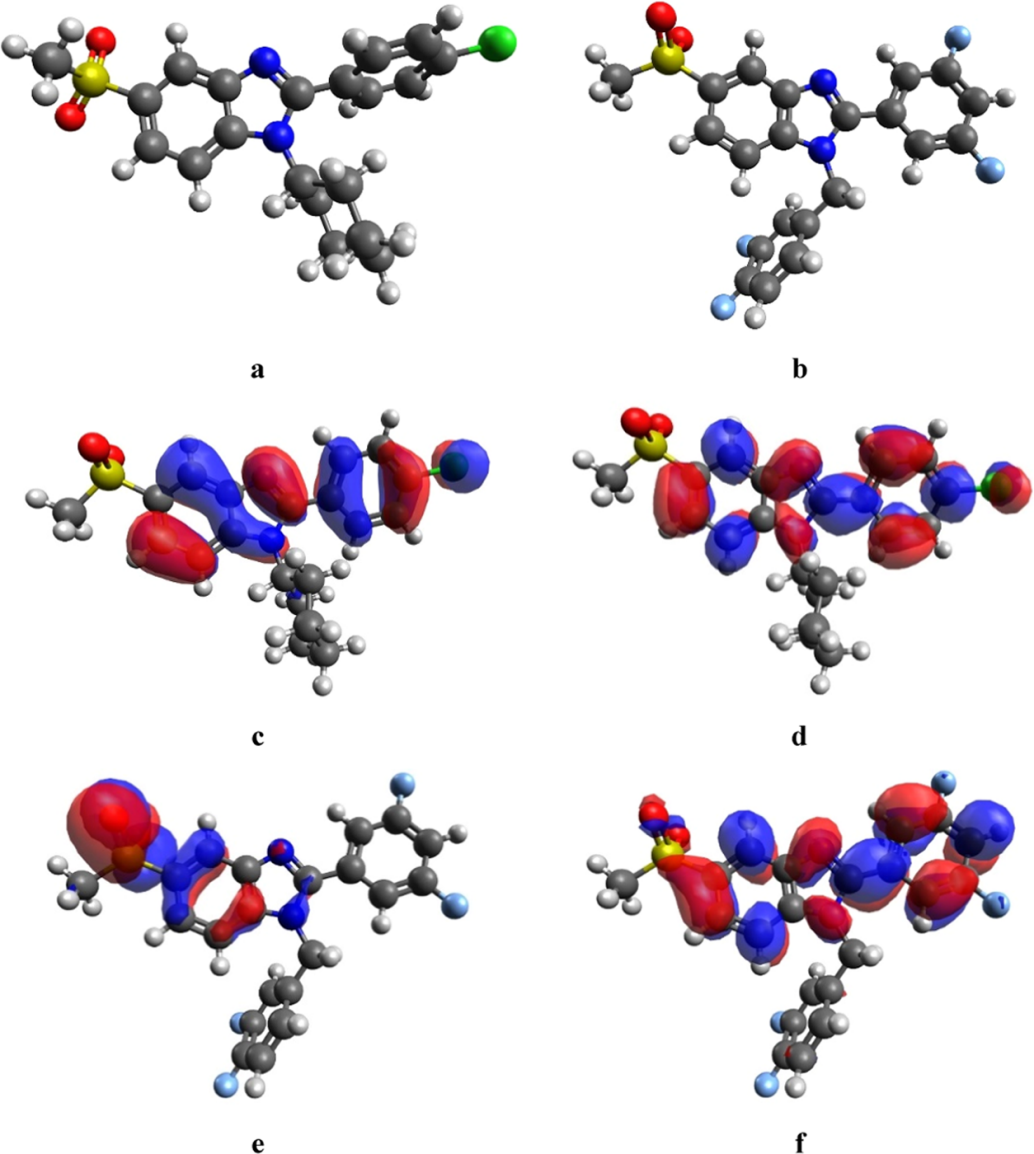
Optimized structures of (a) **23** and (b) **27**. HOMO and LUMO plots of compound **23** (c, d) (total DFT energy = −1893.625517). HOMO and LUMO plots of compound **27** (e, f) (total DFT energy = −1866.7497441335).

DFT analysis of compounds **23** and **27** was performed to provide insights into their electron clustering, chemical stability, and reactivity. For obtaining the molecular orbitals of the active compounds, initial geometric optimization was made using Avogadro software, and the optimized structures are given in Figure 6. The perpendicular conformation of the methylsulfonyl and benzene rings in compound **23** (Figure 6a) was not apparent in **27** (Figure 6b). Yet, cyclohexyl and 3,4-difluorobenzyl moieties both converged in perpendicular conformations. This difference alone may alter the binding characteristics of the compounds which could lead them to irrelevant biotargets. The highest occupied molecular orbital (HOMO) and lowest occupied molecular orbital (LUMO) plots of the active compounds **23** and **27** were given in Figure 6c–6f. These orbitals of a molecule affect the optical and electronic properties, chemical reactivity-stability, and biological activity. HOMO energy is the ability to give electrons, while the LUMO energy is the ability to receive electrons. If the difference between the HOMO and LUMO energy values is high, the reactivity of the compound is low, the stability is therefore higher.^58^ Compound **23** has a HOMO–LUMO gap value of 3.126 eV, which suggests that it is stable. According to Figure 6c, orbitals of **23** are mostly concentrated on the benzimidazole moiety, benzene ring, and chlorine atom, whereas the methylsulfonyl and cyclohexyl groups are not prone to possible reactions. This phenomenon is not altered in the LUMO state of this compound (Figure 6d), which suggests that even in an excited state, methylsulfonyl and cyclohexyl moieties are the stable regions of this compound.

The HOMO–LUMO gap value of **27** was 2.135 eV, suggesting that the general stability is high. The molecular orbitals given in Figure 6e give a peculiar profile for the HOMO state of **27** (Figure 6e) since it shows that the molecular orbitals are concentrated at the sulfonyl moiety of the molecule. This is alarming for the reactivity and stability of the sulfonyl group. Fortunately, this phenomenon changes drastically for the LUMO state, exerting similarity to the LUMO state of compound **23** (Figure 6f). In the LUMO state given in Figure 6f, 3,5-difluorophenyl substituent attached to the second position of benzimidazole becomes surrounded by molecular orbitals, rendering it prone to the reactions. Then again, the σ bond between this group and benzimidazole carbon becomes alarmingly susceptible to reactions. These molecular orbitals’ illustrations provide insights into the molecular reactivity and stability of the active molecules **23** and **27**. Moreover, HOMO–LUMO energy gap values for the compounds **23**–**36** were given in Supporting Table 1. Accordingly, the second active compound **27** exerted the smallest gap (2.135 eV) between the HOMO and LUMO states. However, **23** came in distant fourth place with a gap value of 3.126 eV. The results were positive for rationalizing the greater activity of prominent compounds **23** and **27**.

### 3.7 MEP Analysis

**Figure 7:**
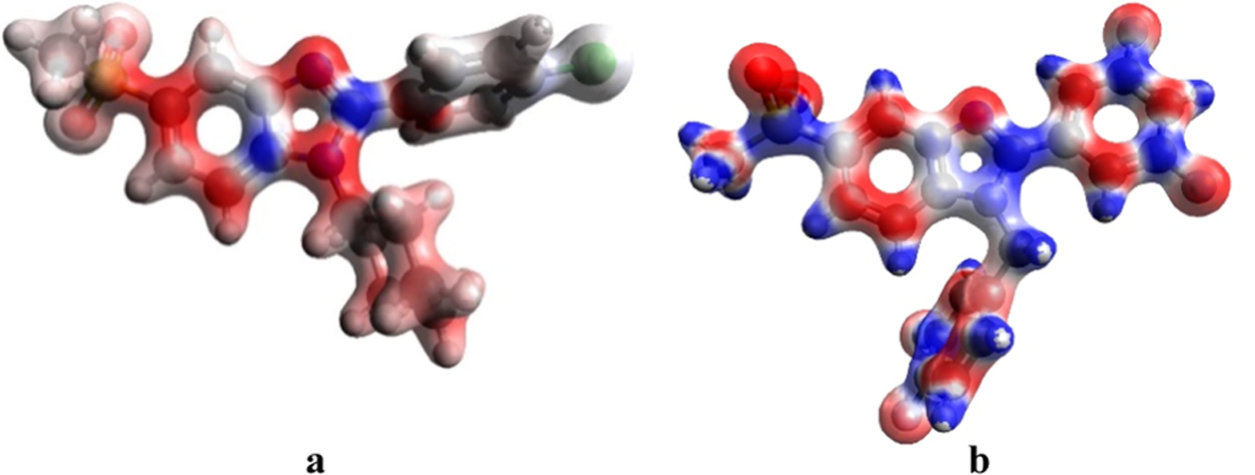
Electrostatic potential maps of compounds (a) **23** and (b) **27**.

The molecular electrostatic potential (MEP) map of a molecule calculated by ORCA software consists of red, blue, and white regions. The region with the red color indicates the negative region (electron-rich), while blue is for the relatively positive region (electron-poor). Electron density and therefore, the chemical reactivity is higher in the red region, whereas the reaction stability is lower in the blue region.^59^ The MEP maps for compounds **23** and **27** are given in Figure 7. Electron densities of the methylsulfonyl group (attached to the fifth position of benzimidazole) and phenyl (attached to the second carbon of benzimidazole) are relatively scarce in **23** (Figure 7a). The nitrogen atoms and 5th–7th carbon atoms in the benzimidazole ring of **23** are prone to electrophilic reactions. Two halogen-substituted phenyl rings attached to the benzimidazole ring influence the benzimidazole ring in a manner that decreases the density of both the first and second atoms (Figure 7b). Herein, the hydrogens attached to this ring turned into electrophilic groups. Fluorine atoms in the ring seem to exert more nucleophilic character compared to chlorine present in **23** (Figure 7a). Due to their electronegativity, they withdraw electrons from both the first and second atoms of benzimidazole. This effect is not apparent for compound **23** (Figure 7a). These features of **27** may be responsible for its bioactivity on receptors, whereas the driving force of activity of **23** would most likely be benzimidazole and the attached cyclohexyl ring.

### 3.8 ADME Estimation

**Figure 8:**
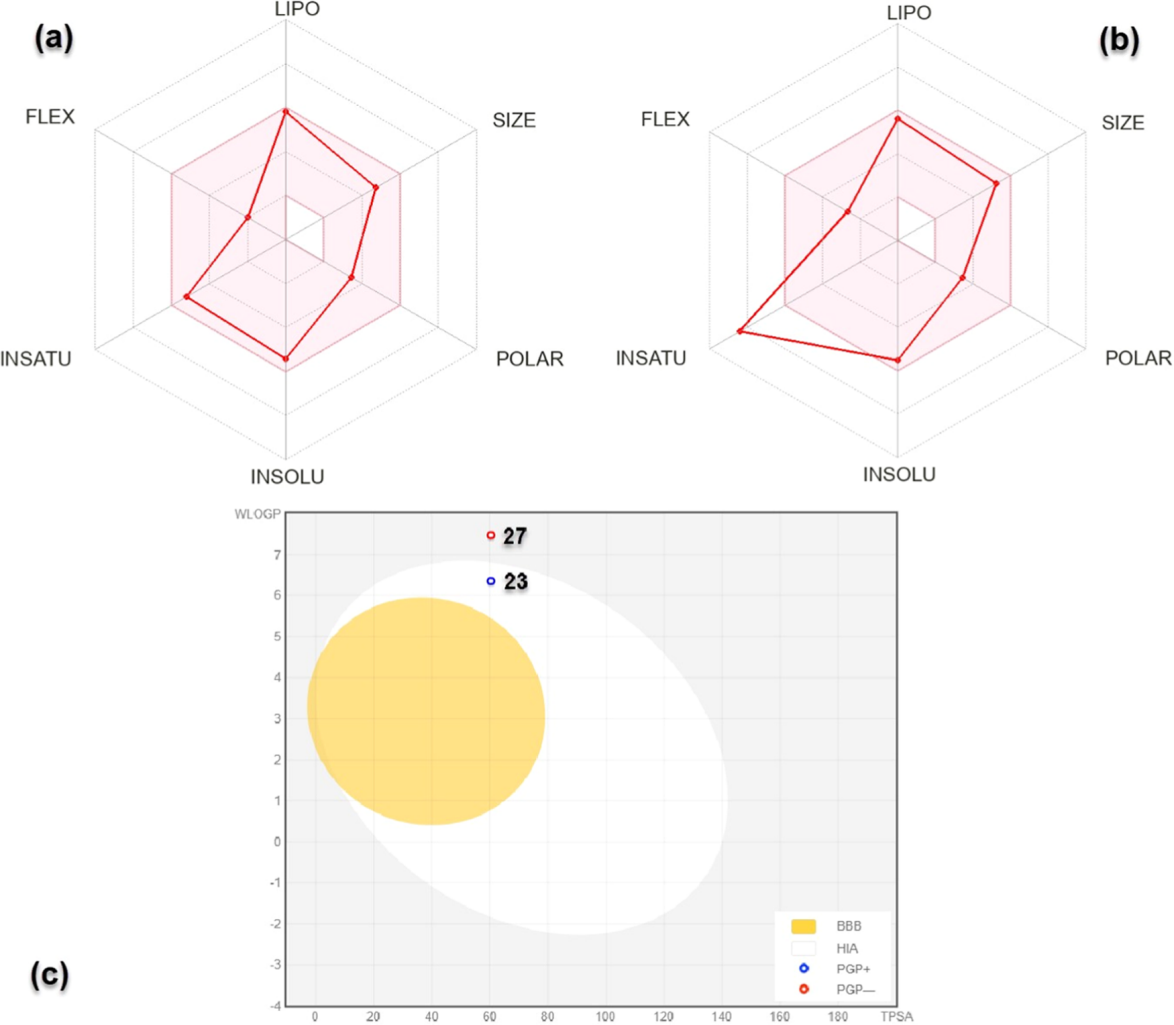
ADME radar plots of (a) **23** and (b) **27** with (c) boiled egg model obtained from the SwissADME server.

The ADME parameters, including lipophilicity, BBB penetration, aqueous solubility, and CYP450 inhibition, were evaluated using the SwissADME tool. SwissADME is a free, user-friendly web-based computational tool employed for the assessment of pharmacokinetics, drug-likeness, and medicinal chemistry friendliness of small molecules.^39^ To predict if our synthesized benzimidazole derivatives can be successfully developed into conventional drugs, we evaluated the pharmacokinetic and drug-like parameters of compounds **23**–**36**. Again, for this analysis, vincristine was used as a standard. Data obtained from the program are tabulated as shown in Table 4. Although several compounds from studies show remarkable biological activities, many have failed to excel in the drug development journey, partly due to their poor pharmacokinetic credentials, including adsorption, distribution, metabolism, and excretion.^60^ In the realm of drug development, *in silico* ADME screening serves as a valuable tool for identifying the most promising compounds and mitigating the potential for drug attrition at the advanced stages of drug research.^61^ The *n*-octanol/water partition coefficient (log Po/*w*) is a fundamental physicochemical parameter for drug discovery, design, and development.^62^ The log Po/*w* is a typical quantitative and classical descriptor of the lipophilicity of a compound and this parameter by extension provides insight into their bioavailability and toxicity. To enhance the predictive accuracy of this descriptor, the consensus log Po/*w* is usually adopted. The consensus log Po/*w* is the arithmetic mean of the values predicted by the five proposed computational methods for log Po/*w* estimation. These predictive models include XLOGP3, WLOGP, MLOGP, SILICOS-IT, and iLOGP.^39^ The consensus log Po/*w* values for the synthesized derivatives range between 2.10 and 4.96. Compound **27** had the highest consensus log Po/*w* value (Table 4), and this suggests its accumulation in tissues would be much higher leading to increased toxicity. Permeation through BBB is undesirable for the synthesized derivatives with anticancer properties as this parameter would influence the cytotoxicity of the central nervous system (CNS).^63^ Except for compound **30**, all our synthesized derivatives are predicted to be impermeable to BBB. *P*-glycoprotein (*P*-gp) is an important protein present on cell membranes responsible for the transport of small molecules. It is suggested to be the most significant member among the ATP-binding cassette (ABC) transporters.^64^*P*-gp also protects the CNS from xenobiotics.^65^ We envisaged our compounds **23**–**26** could be substrates for this protein. However, only vincristine and compounds **23**–**26** are predicted to be substrates of *P*-gp. According to the ADME radar plots obtained from the SwissADME program, compound **27** was outside the insaturation (INSATU) limit (Figure 8b).

**Table 4:** SwissADME-Predicted Physicochemical and Drug-likeness Properties of Compounds **23**–**36**

comp.	consensus log Po/*w*	BBB^a^ permeation	substrate to P-gp^a^	CYP450 inhibition (out of 5)	TPSA (Å^2^)	num. of rotatable bonds	num. of H-bond acceptor	num. of H-bond donor	Lipinski^a^ score	Veber^a^ score
**23**	4.46	0	1	4	60.34	3	3	0	1	1
**24**	4.53	0	1	2	60.34	3	5	0	1	1
**25**	4.80	0	1	3	60.34	4	3	0	1	1
**26**	4.87	0	1	3	60.34	4	5	0	1	1
**27**	4.96	0	0	3	60.34	4	7	0	1	1
**28**	4.95	0	0	3	60.34	4	7	0	1	1
**29**	2.10	0	0	1	103.43	5	4	1	1	1
**30**	3.23	1	0	3	60.34	3	4	0	1	1
**31**	4.24	0	0	5	60.34	6	4	0	1	1
**32**	3.52	0	0	4	89.44	8	4	1	1	1
**33**	4.42	0	0	2	86.64	6	7	0	1	1
**34**	4.46	0	0	5	60.34	6	3	0	1	1
**35**	4.67	0	0	4	60.34	4	6	0	1	1
**36**	3.75	0	0	3	89.44	6	5	1	1	1
VCR	3.40	0	1	1	171.17	11	12	3	0	0

a Satisfying the parameter in the column is denoted by 1, while not satisfying the parameter is denoted by 0.

Both compounds were suitable for other radar plot parameters (e.g., Figure 8a for compound **23**). Based on the BOILED-Egg depiction, compound **23** is likely to get reabsorbed in the gastrointestinal tract, whereas **27** may not. Both compounds were impermeable to the BBB (Figure 8c). Several drug molecules are mainly eliminated via CYP450-mediated biotransformation. Hence, drug interactions resulting from drug metabolism inhibition occur in several molecules.^66^ Cytochrome-P450 enzyme inhibition was also calculated to discern candidates with propensities for drug–drug interactions. Based on the calculated values of CYP450 enzyme inhibition, compounds **31** and **34** attained the maximum score of 5 and are thus predicted to be potential inhibitors of all of the CYP450 five major isoenzymes including CYP1A2, CYP2C19, CYP2C9, CYP2D6, and CYP3A4. This portends the synthesized derivatives can elicit several drug interactions that may lead to drug toxicities, diminished pharmacological effects, and adverse drug reactions. This pharmacokinetic interaction can occur with drug molecules that have substrate affinity with the isoenzymes. Inhibition of CYP isoenzymes is one of the key determinants of pharmacokinetics-related drug–drug interactions. This leads to toxic or other undesired adverse effects due to decreased elimination and buildup of the drug molecules or their metabolites.^67^ The Lipinski rule of five (RO5) is a fundamental chemo-informatics filter which states that an orally bioactive drug must fulfill at least 4 of 5 criteria namely: molecular weight <500 g/mol, hydrogen bond donor ≤5, hydrogen bond acceptor ≤10, and octanol–water partition coefficient (log *P*) < 5 to achieve aqueous solubility or intestinal permeability.^68^

Based on the values of molecular descriptors examined, all our compounds passed the Lipinski filter as denoted by a score of 1. The standard drug vincristine failed to comply with the Lipinski filter as it had two violations of the rule: molecular weight greater than 500 (824.96); and the number of its hydrogen bond acceptor exceeds the threshold of 10 (12). Similarly, vincristine failed the Veber filter based on the values of rotatable bonds (11) and topological polar surface area (TPSA) (171.17 Å^2^). According to Veber’s two-criteria rule, for compounds to adhere to optimal bioavailability, they should have a TPSA ≤ 140 Å^2^ and rotatable bonds ≤10.^69^ The number of rotatable bonds serves as a measure of molecular flexibility, a crucial factor influencing the oral bioavailability of a compound. Hence, a flexible molecule indicates it is less orally active. Furthermore, there has been a proposition that the number of hydrogen bonding groups could be substituted with TPSA as a factor in the computation of percentage absorption.^70^ Compounds with TPSA ≤ 140 Å^2^ and ≤10 rotatable bonds are expected to demonstrate high oral bioavailability.^69^ With the exception of standard drug vincristine, all our synthesized compounds have TPSA (60.34–103.43 Å^2^) and the number of rotatable bonds (3–8) within the normal ranges of the Veber criteria. Interestingly, all of the synthesized derivatives comply with Lipinski and Veber filters, thus making them suitable candidates that may warrant further computational studies for their development.

## 4 Conclusions

The search for novel benzimidazole compounds is a key focus in medicinal chemistry research. This is very crucial to providing diverse therapeutic options for the treatment of diseases. This study reported the synthesis of a series of new alkylsulfonyl 1*H*-benzo[*d*]imidazole derivatives. Based on the IC_50_ values, compounds **23** and **27** were found to be the two most potent cytotoxic derivatives against MCF-7 breast cancer cells. The result of the cytotoxic activity corroborated the gene expression studies, wherein the two compounds downregulated the expression of the antiapoptotic gene BCL-2 by 128- and 256-fold, respectively. Furthermore, molecular docking suggests the presence of halogens and sulfonyl groups in the compounds played a significant role in their hydrogen bond interaction with DNA bases, leading to higher cytotoxic activity and good docking profile. Molecular dynamics analysis also led credence to the docking results in which the interaction and accommodation of compounds **23** and **27** were stable for 200 ns. The DFT findings showed the electron density for compound **27** was higher in the sulfonyl group and fluorine atoms. On the other hand, ADME radar plots of **23** indicated molecular orbitals clustered over the chlorine atom, thus leaving the sulfonyl group electron deficient. The HOMO–LUMO gap was lowest at **27**, which is evident by the high activity of this derivative. The outcome of ADME studies suggests that the synthesized derivatives are suitable drug candidates that could be explored in further computational studies to improve their therapeutic potential as potential Bcl-2 inhibitors with anticancer activity.

## Supporting Information Available

Brief description of the genes studied; spectra (^1^H NMR, ^13^C NMR, MS); HOMO–LUMO gap values of all compounds; and molecular dynamics simulation animation of compounds **23** and **27** (PDF)
