# Supplementary material for: Synthesis, Anticancer Activity, and In Silico Modeling of Alkylsulfonyl Benzimidazole Derivatives: Unveiling Potent Bcl-2 Inhibitors for Breast Cancer
Source: ACS Omega. 2024 Feb 14;9(8):9547–63. doi: 10.1021/acsomega.3c09411 (PMC10905736; doi:10.1021/acsomega.3c09411)
Supplement: Supplementary file 1 — ao3c09411_si_001.pdf [file ao3c09411_si_001.pdf]

Supporting Information

# Synthesis, Anticancer Activity and *In Silico* Modelling of Alkylsulfonyl Benzimidazole Derivatives: Unveiling Potent Bcl-2 Inhibitors for Breast Cancer

Yemna Abbade[1,2], Mehmet Murat Kisla[1,2], Mohammed Al-Kassim Hassan[1,2,3], Ismail Celik[4], Tugba Somay Dogan[5], Pelin Mutlu[6], Zeynep Ates-Alagoz[*1]

[1]*Department of Pharmaceutical Chemistry, Faculty of Pharmacy, Ankara University, 06100, Ankara, Turkey*

[2]*Graduate School of Health Sciences, Ankara University, 06110 Ankara, Turkey*

[3]*Department of Pharmaceutical and Medicinal Chemistry, Faculty of Pharmaceutical Sciences, Bayero University, P.M.B 3011 Kano, Nigeria*

[4]*Department of Pharmaceutical Chemistry, Faculty of Pharmacy, Erciyes University, 38039, Kayseri, Turkey*

[5]*Central Laboratory, Molecular Biology and Biotechnology R&D Center, Middle East Technical University, 06800, Ankara, Turkey*

[6]*Department of Biotechnology, Biotechnology Institute, Ankara University, 06135, Ankara, Turkey*

***Corresponding author**

*Email: zates@pharmacy.ankara.edu.tr*

**CONTENT**

1. Brief description of genes examined in gene expression analyses……………………………S2-S3
2. ESI-MS, $^{1}$H NMR and $^{13}$C NMR spectra of compounds **23-36** (S1-S35) ………..………….S4-S21
3. Alignment of Bcl-2 co-ligand venetoclax and its re-docked conformer (S36)……………….S22
4. Table of HOMO-LUMO gap values for compounds **23-36** (S37) …………………………...S23

## 1. Brief description of genes examined in gene expression analyses

**ABCC1:** The protein encoded by this gene is a member of the superfamily of ATP-binding cassette (ABC) transporters. ABC proteins transport various molecules across extra-and intra-cellular membranes. ABC genes are divided into seven distinct subfamilies (ABC1, MDR/TAP, MRP, ALD, OABP, GCN20, White). This full transporter is a member of the MRP subfamily which is involved in multi-drug resistance. This protein functions as a multispecific organic anion transporter, with oxidized glutathione, cysteinyl leukotrienes, and activated aflatoxin B1 as substrates.

**TUBD1:** Predicted to enable GTP binding activity. Predicted to be a structural constituent of cytoskeleton. Predicted to be involved in microtubule cytoskeleton organization; mitotic cell cycle; and positive regulation of smoothened signaling pathway.

**MAP7:** The product of this gene is a microtubule-associated protein that is predominantly expressed in cells of epithelial origin. Microtubule-associated proteins are thought to be involved in microtubule dynamics, which is essential for cell polarization and differentiation. This protein has been shown to be able to stabilize microtubules and may serve to modulate microtubule functions.

**MAP4:** The protein encoded by this gene is a major non-neuronal microtubule-associated protein. This protein contains a domain similar to the microtubule-binding domains of neuronal microtubule-associated protein (MAP2) and microtubule-associated protein tau (MAPT/TAU). This protein promotes microtubule assembly and has been shown to counteract destabilization of interphase microtubule catastrophe promotion. Cyclin B was found to interact with this protein, which targets cell division cycle 2 (CDC2) kinase to microtubules. The phosphorylation of this protein affects microtubule properties and cell cycle progression.

**ABCG1:** The protein encoded by this gene is a member of the superfamily of ATP-binding cassette (ABC) transporters. ABC proteins transport various molecules across extra- and intra-cellular membranes. ABC genes are divided into seven distinct subfamilies (ABC1, MDR/TAP, MRP, ALD, OABP, GCN20, White). This protein is a member of the White subfamily. It is involved in macrophage cholesterol and phospholipids transport and may regulate cellular lipid homeostasis in other cell types.

**ESR1:** The protein encoded by this gene regulates the transcription of many estrogen-inducible genes that play a role in growth, metabolism, sexual development, gestation, and other reproductive functions and is expressed in many non-reproductive tissues. The receptor encoded by this gene plays a key role in breast cancer, endometrial cancer, and osteoporosis.

**BIRC3:** This gene encodes a member of the IAP family of proteins that inhibit apoptosis by binding to tumor necrosis factor receptor-associated factors TRAF1 and TRAF2, probably by interfering with activation of ICE-like proteases.

**STAT1:** In response to cytokines and growth factors, STAT family members are phosphorylated by the receptor associated kinases, and then form homo- or heterodimers that translocate to the cell nucleus where they act as transcription activators. The protein encoded by this gene can be activated by various ligands including interferon-alpha, interferon-gamma, EGF, PDGF and IL6. This protein mediates the expression of a variety of genes, which is thought to be important for cell viability in response to different cell stimuli and pathogens.

**MYC:** This gene is a proto-oncogene and encodes a nuclear phosphoprotein that plays a role in cell cycle progression, apoptosis and cellular transformation. Amplification of this gene is frequently observed in numerous human cancers.

**PDCD10:** This gene encodes an evolutionarily conserved protein associated with cell apoptosis. The protein interacts with the serine/threonine protein kinase MST4 to modulate the extracellular signal-regulated kinase (ERK) pathway.

**BCL-2:** This gene encodes an integral outer mitochondrial membrane protein that blocks the apoptotic death of some cells such as lymphocytes. Constitutive expression of BCL2, such as in the case of translocation of BCL2 to Ig heavy chain locus, is thought to be the cause of follicular lymphoma.

**MCL-1:** This gene encodes an anti-apoptotic protein, which is a member of the Bcl-2 family.

**TNFRSF6B:** This gene belongs to the tumor necrosis factor receptor superfamily. The encoded protein is postulated to play a regulatory role in suppressing FasL- and LIGHT-mediated cell death. It acts as a decoy receptor that competes with death receptors for ligand binding. Over-expression of this gene has been noted in gastrointestinal tract tumors.

**TP53:** This gene encodes a tumor suppressor protein containing transcriptional activation, DNA binding, and oligomerization domains. The encoded protein responds to diverse cellular stresses to regulate expression of target genes, thereby inducing cell cycle arrest, apoptosis, senescence, DNA repair, or changes in metabolism. Mutations in this gene are associated with a variety of human cancers.

**RB1:** The protein encoded by this gene is a negative regulator of the cell cycle and was the first tumor suppressor gene found.

**CDK4:** The protein encoded by this gene is a member of the Ser/Thr protein kinase family. This protein is highly similar to the gene products of S. cerevisiae cdc28 and S. pombe cdc2. It is a catalytic subunit of the protein kinase complex that is important for cell cycle G1 phase progression. The activity of this kinase is restricted to the G1-S phase, which is controlled by the regulatory subunits D-type cyclins and CDK inhibitor p16(INK4a). This kinase was shown to be responsible for the phosphorylation of retinoblastoma gene product (Rb) .

**ABL1:** This gene is a protooncogene that encodes a protein tyrosine kinase involved in a variety of cellular processes, including cell division, adhesion, differentiation, and response to stress. The ubiquitously expressed protein has DNA-binding activity that is regulated by CDC2-mediated phosphorylation, suggesting a cell cycle function.

**BRCA1:** This gene encodes a 190 kD nuclear phosphoprotein that plays a role in maintaining genomic stability, and it also acts as a tumor suppressor.

**FAS:** The protein encoded by this gene is a member of the TNF-receptor superfamily. This receptor contains a death domain. It has been shown to play a central role in the physiological regulation of programmed cell death and has been implicated in the pathogenesis of various malignancies and diseases of the immune system. The interaction of this receptor with its ligand allows the formation of a death-inducing signaling complex that includes Fas-associated death domain protein (FADD), caspase 8, and caspase 10.

**CASP9:** This gene encodes a member of the cysteine-aspartic acid protease (caspase) family. Sequential activation of caspases plays a central role in the execution-phase of cell apoptosis.

**BRIP1:** The protein encoded by this gene is a member of the RecQ DEAH helicase family and interacts with the BRCT repeats of breast cancer, type 1 (BRCA1). The bound complex is important in the normal double-strand break repair function of breast cancer, type 1 (BRCA1).

**CDK6:** The protein encoded by this gene is a member of the CMGC family of serine/threonine protein kinases. This kinase is a catalytic subunit of the protein kinase complex that is important for cell cycle G1 phase progression and G1/S transition.

**MAP1B:** This gene encodes a protein that belongs to the microtubule-associated protein family. The proteins of this family are thought to be involved in microtubule assembly, which is an essential step in neurogenesis.

**PDCD6:** This gene encodes a calcium-binding protein belonging to the penta-EF-hand protein family. Calcium binding is important for homodimerization and for conformational changes required for binding to other protein partners. This gene product participates in T cell receptor-, Fas-, and glucocorticoid-induced programmed cell death.

## 2. ESI-MS, ¹H NMR, and ¹³C NMR spectra of compounds 23-36 (S1-S35)

2-(4-Chlorophenyl)-1-cyclohexyl-5-(methylsulfonyl)-1*H*-benzo[*d*]imidazole (**23**)

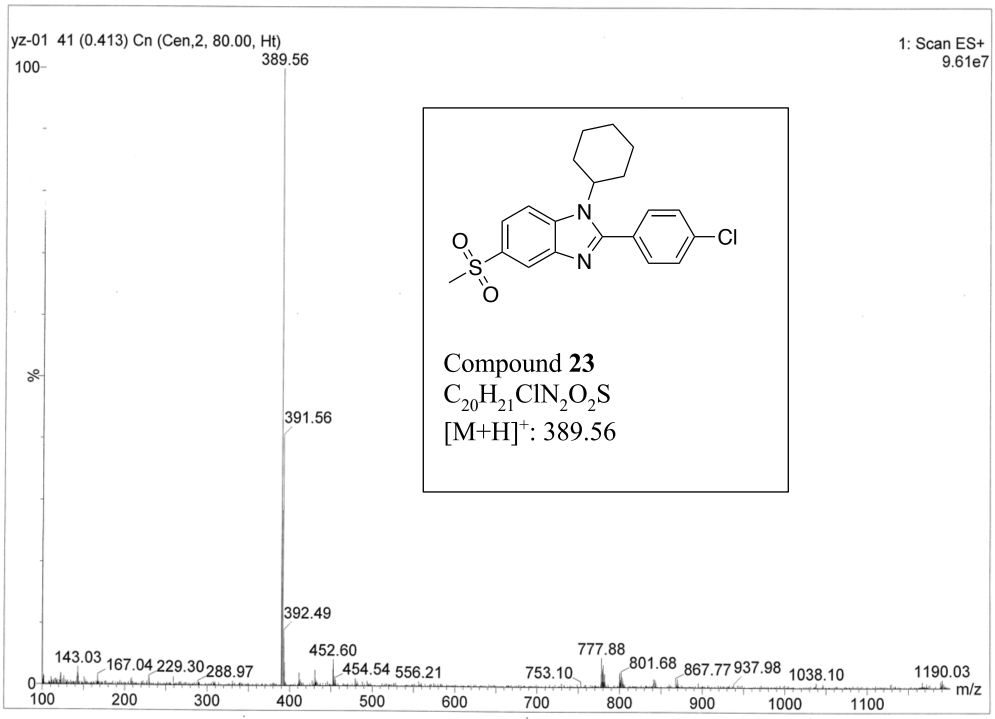


**Figure S1.** ESI-MS spectrum of compound **23**

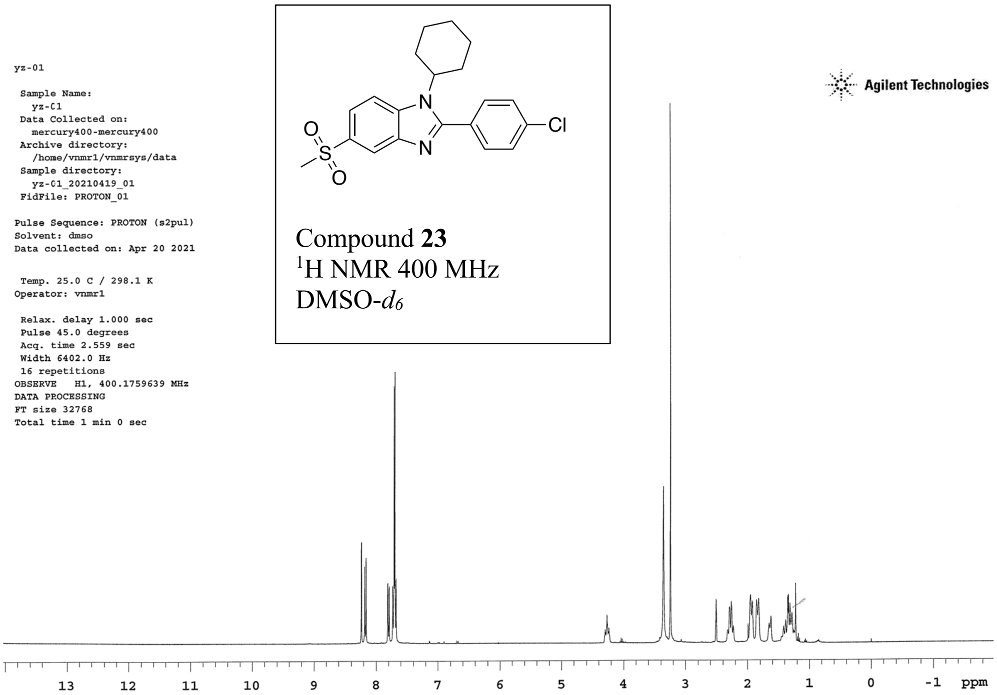


**Figure S2.** ¹H NMR spectrum of compound **23**

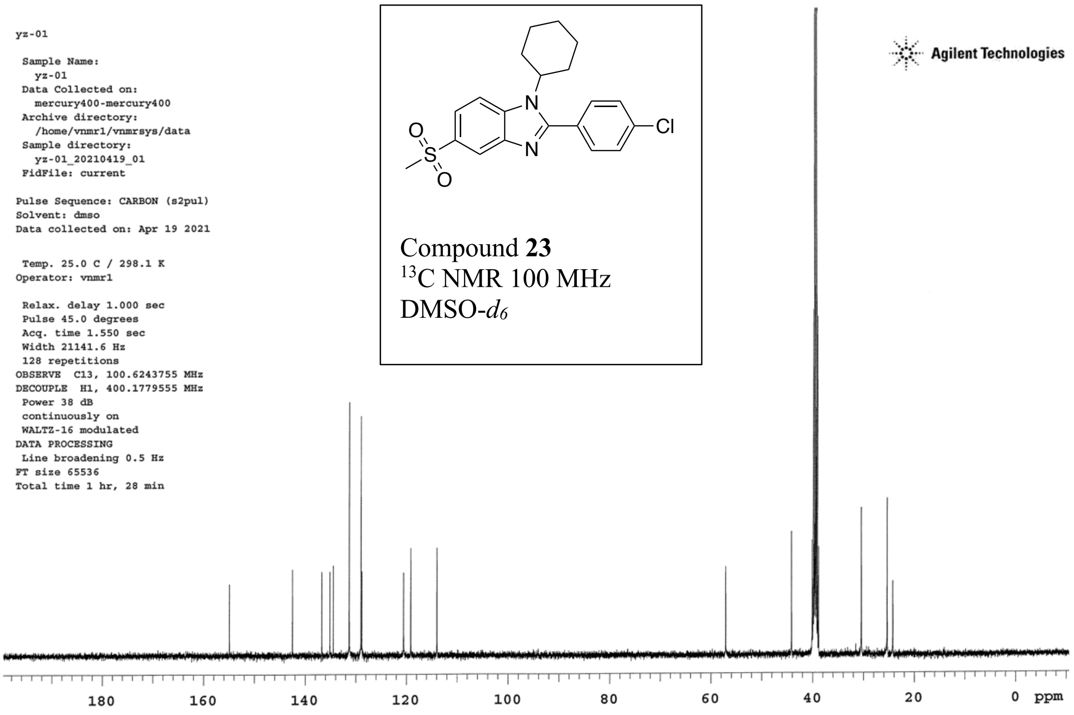


**Figure S3.** $^{13}$C NMR spectrum of compound **23**

1-Cyclohexyl-2-(3,4-difluorophenyl)-5-(methylsulfonyl)–1*H*-benzo[*d*]imidazole (**24**)

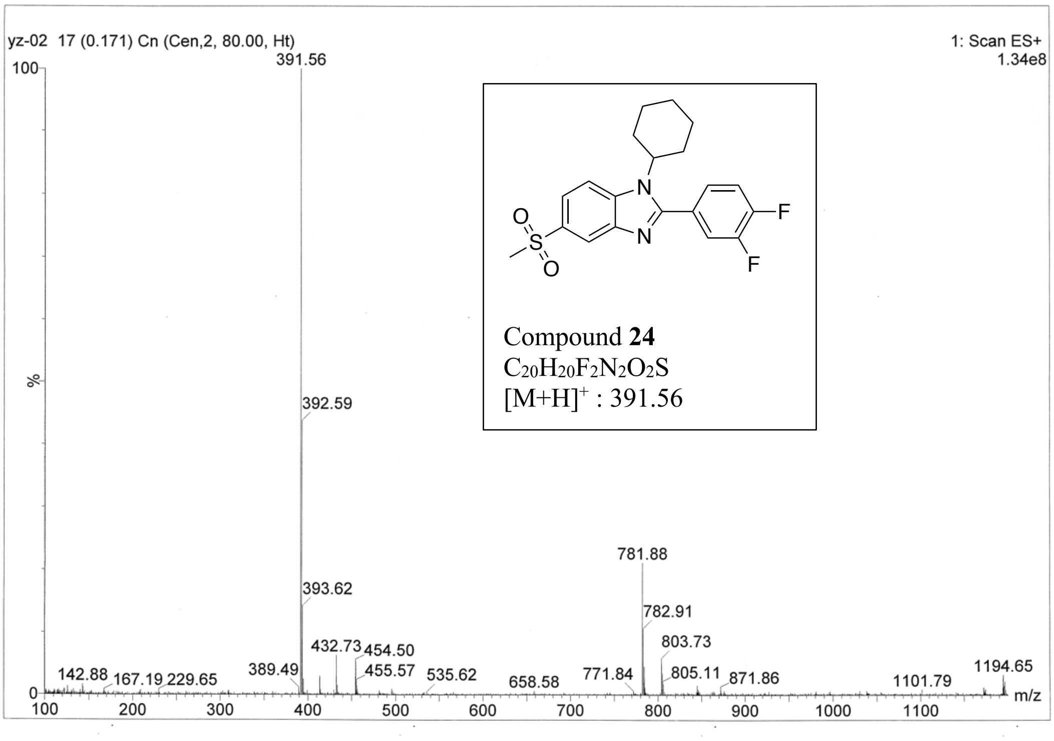


**Figure S4**. ESI-MS spectrum of compound **24**

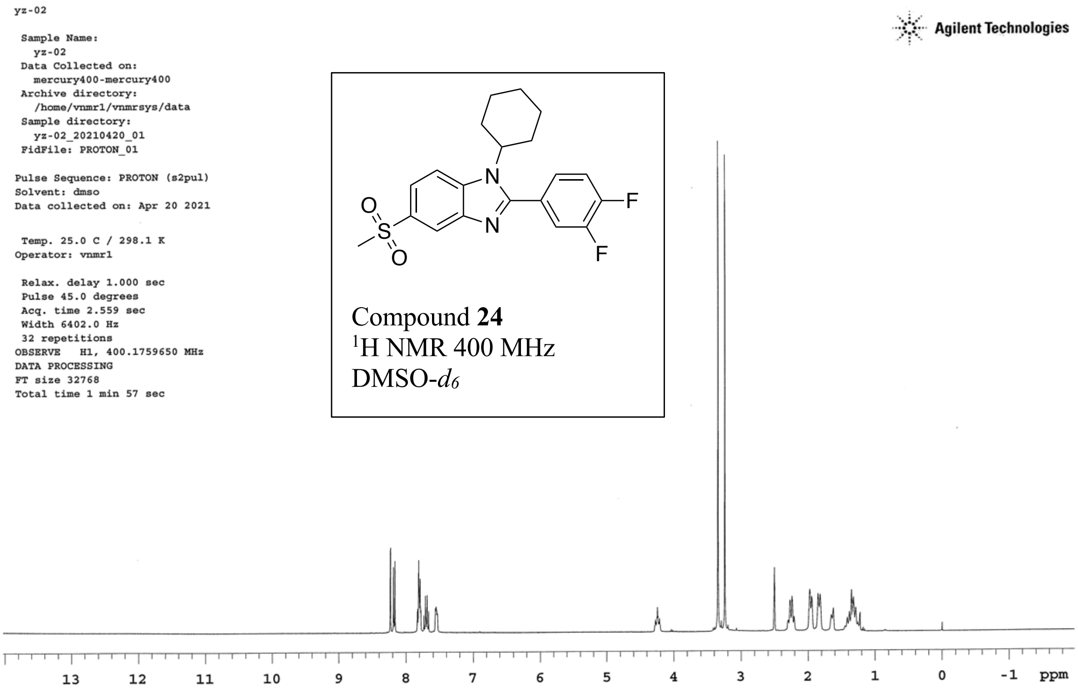


**Figure S5.** $^{1}$H NMR spectrum of compound **24**

2-(4-Chlorophenyl)-1-cyclohexyl-5-(ethylsulfonyl)-1*H*-benzo[*d*]imidazole (**25**)

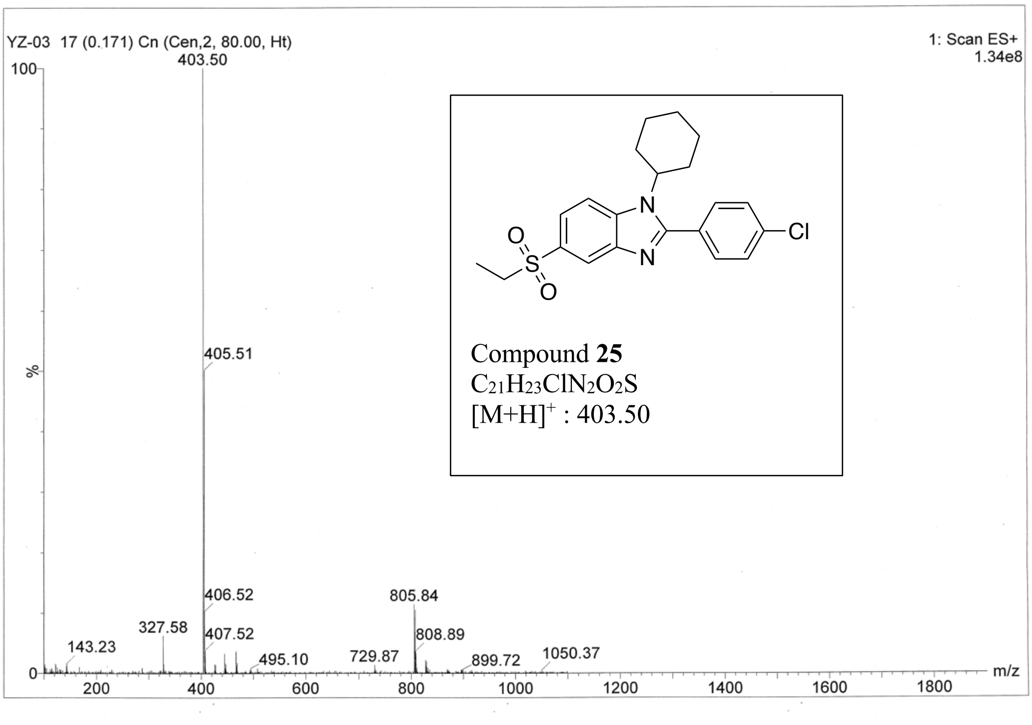


**Figure S6**. ESI-MS spectrum of compound **25**

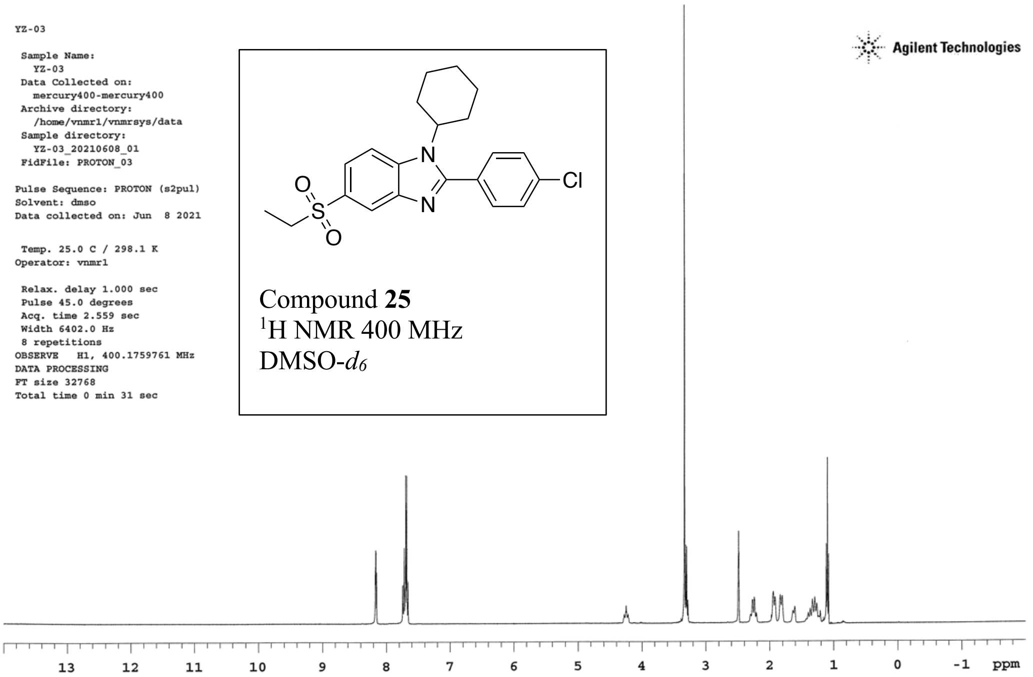


**Figure S7.** $^{1}$H NMR spectrum of compound **25**

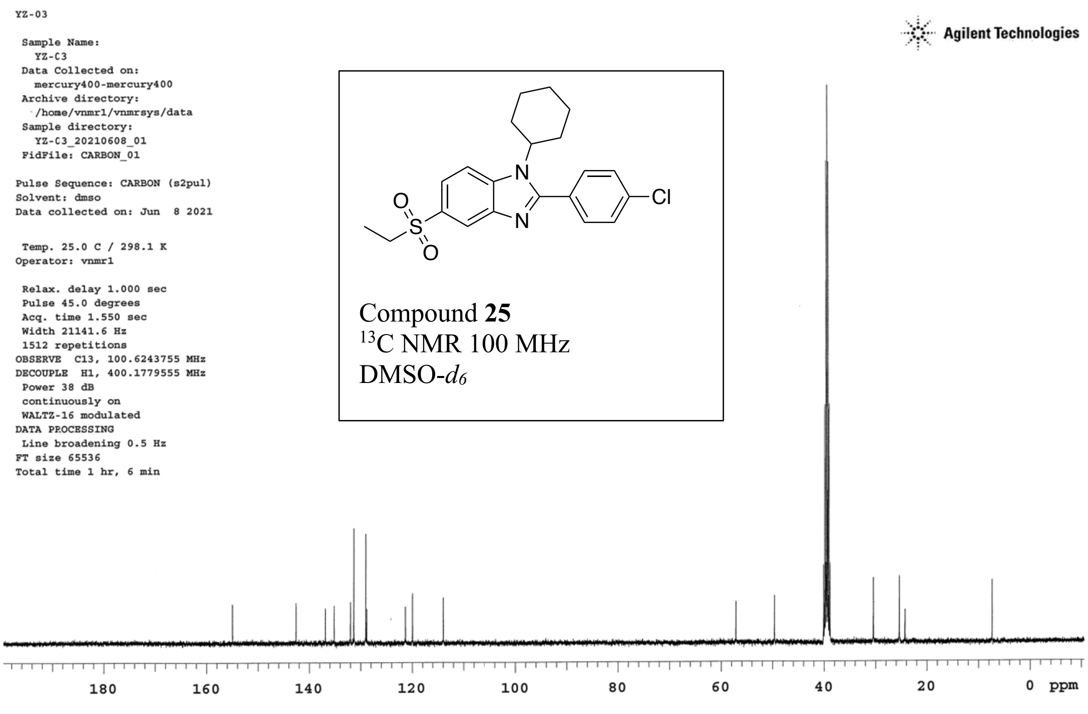


**Figure S8**. $^{13}$C NMR spectrum of compound **25**

1-Cyclohexyl-2-(3,4-difluorophenyl)-5-(ethylsulfonyl)-1*H*-benzo[*d*]imidazole (**26**)

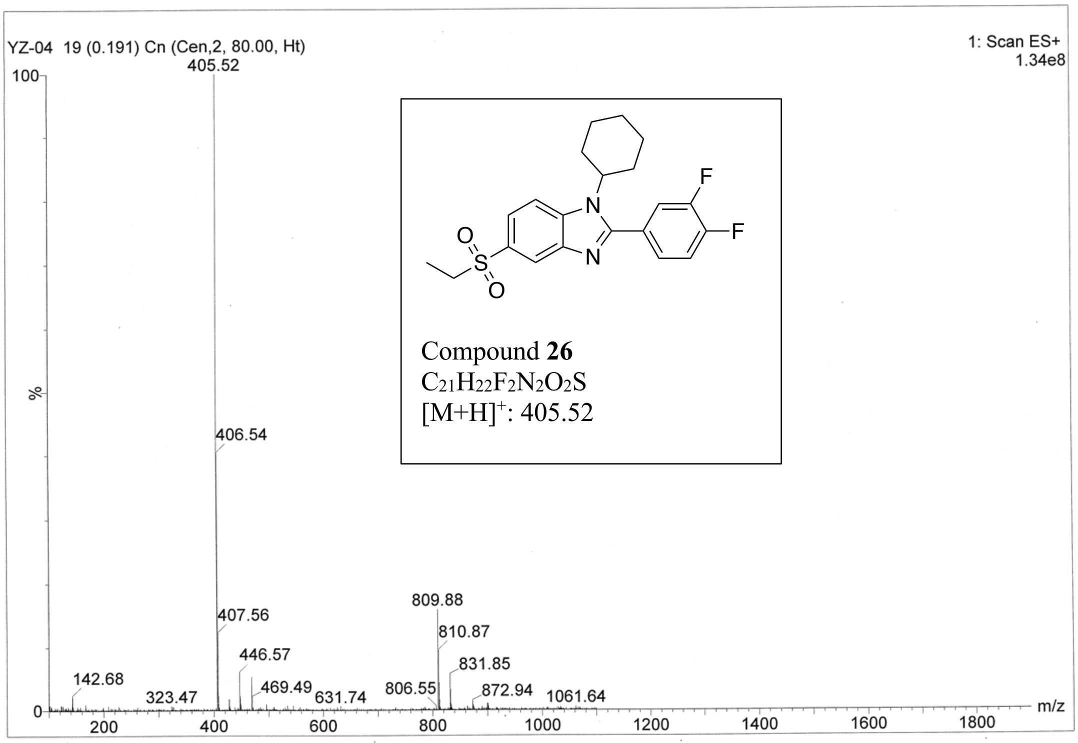


**Figure S9.** ESI-MS spectrum of compound **26**

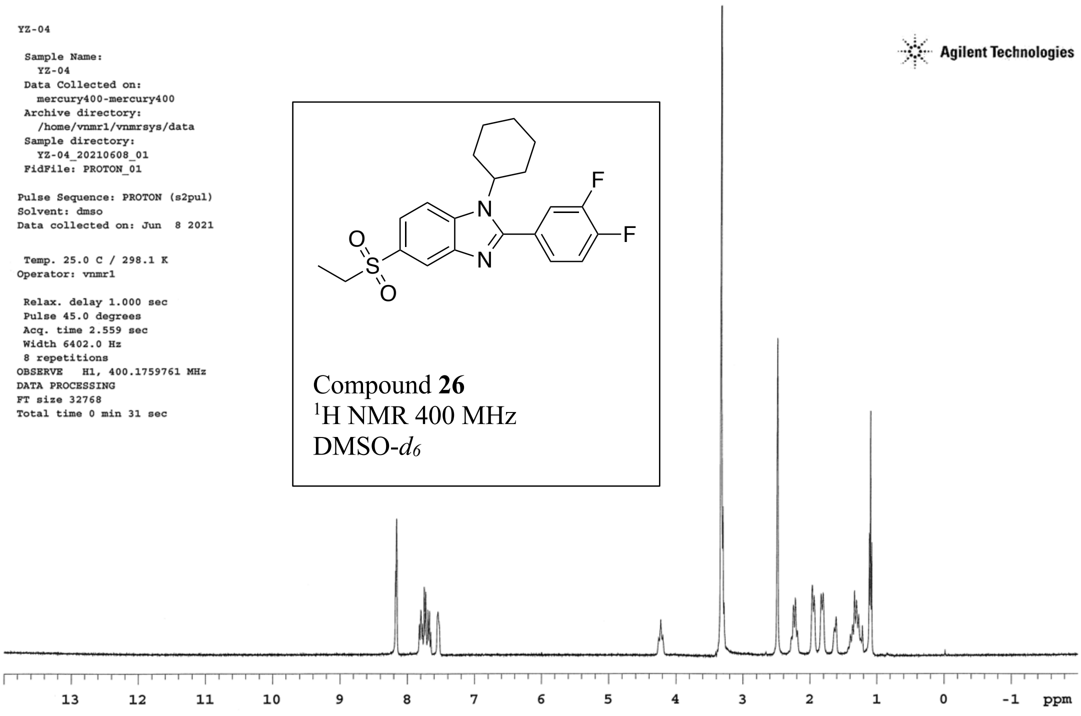


**Figure S10.** $^{1}$H NMR spectrum of compound **26**

1-(3,4-Difluorobenzyl)-2-(3,5-difluorophenyl)-5-(methylsulfonyl)-1*H*-benzo[*d*]imidazole (**27**)

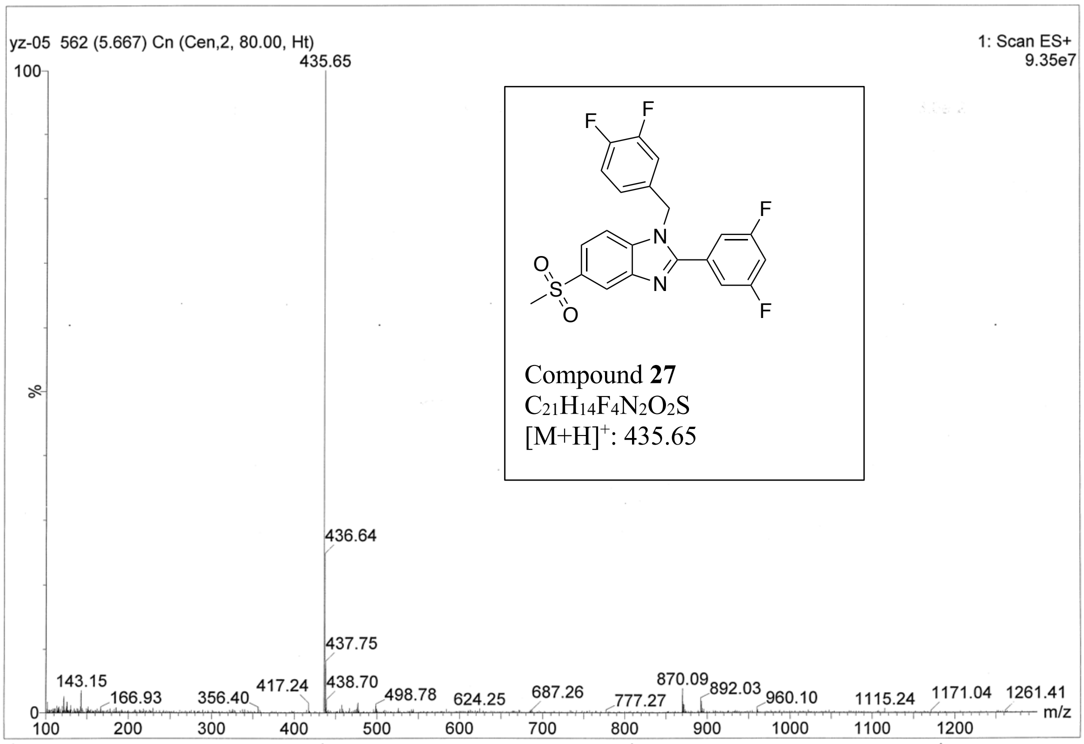


**Figure S11.** ESI-MS spectrum of compound **27**

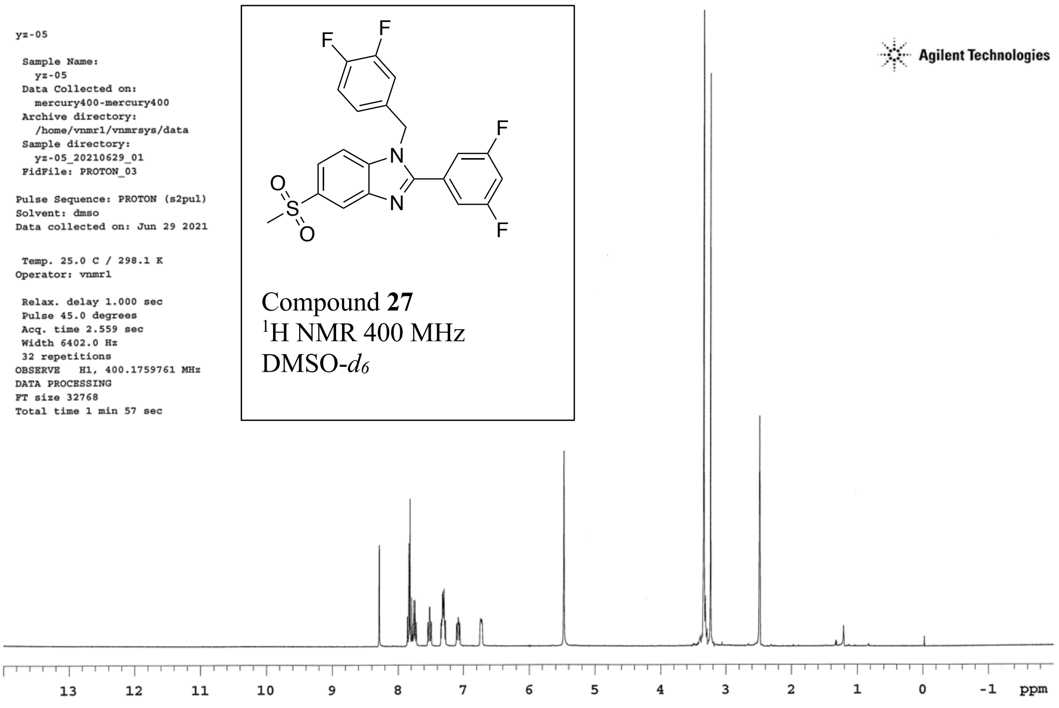


**Figure S12**. $^{1}$H NMR spectrum of compound **27**

1-(3,4-Difluorobenzyl)-2-(2,5-difluorophenyl)-5-(methylsulfonyl)-1*H*-benzo[*d*]imidazole (**28**)

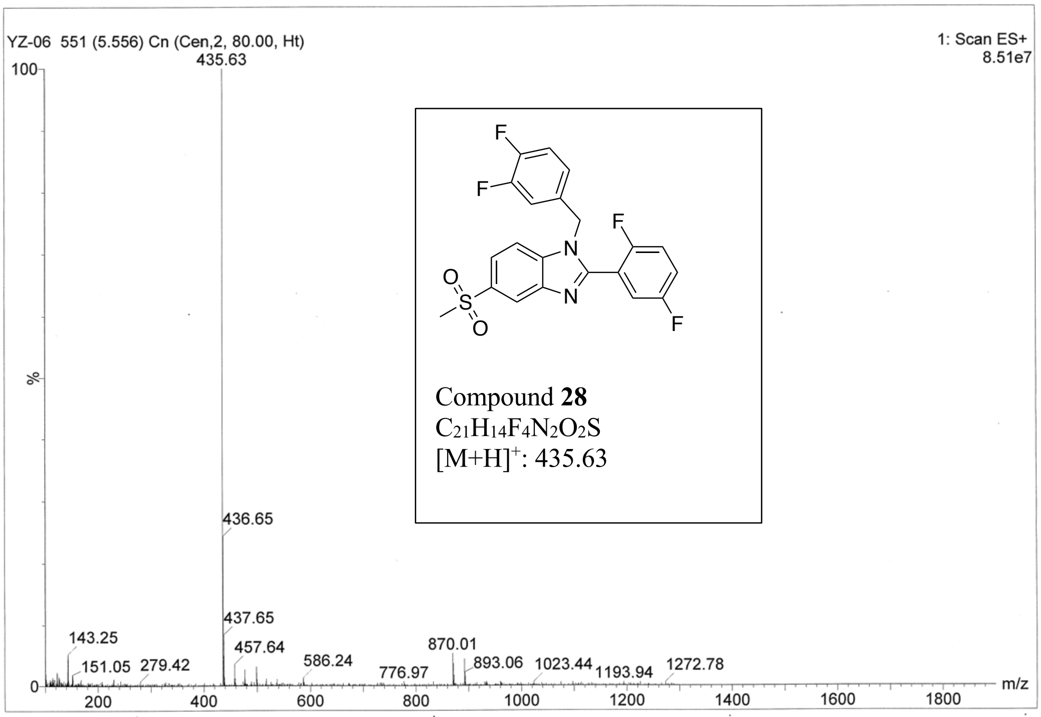


**Figure S13.** ESI-MS spectrum of compound **28**

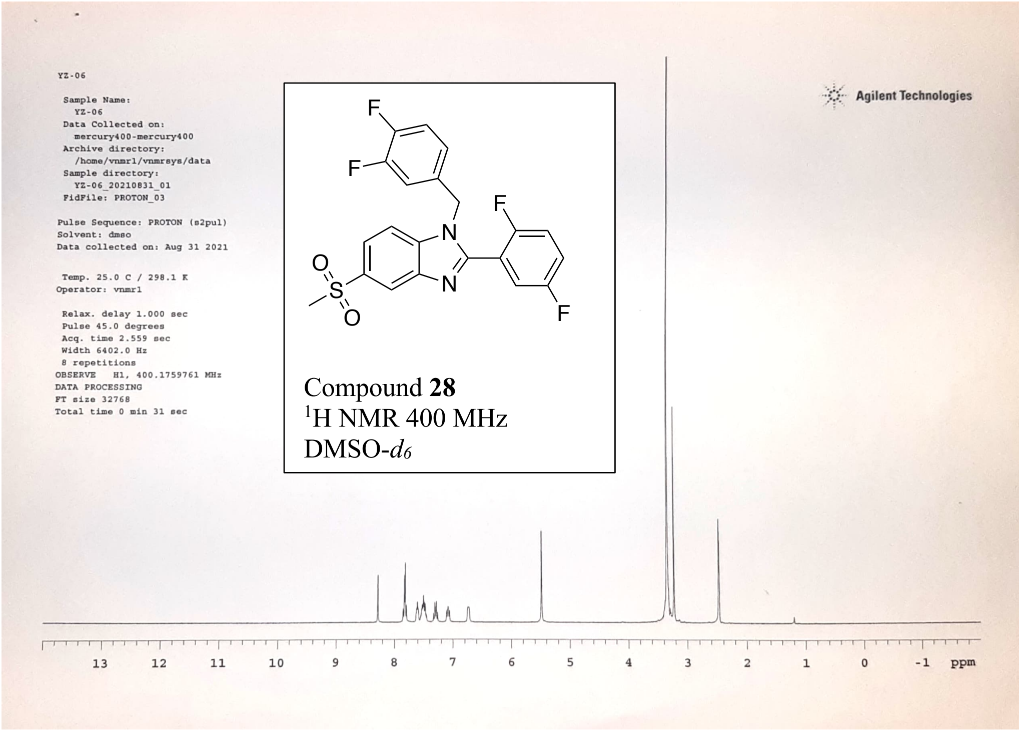


**Figure S14.** $^1$H NMR spectrum of compound **28**

*N*-(4-(1-ethyl-5-(methylsulfonyl)-1*H*-benzo[*d*]imidazol-2-yl)phenyl)acetamide (**29**)

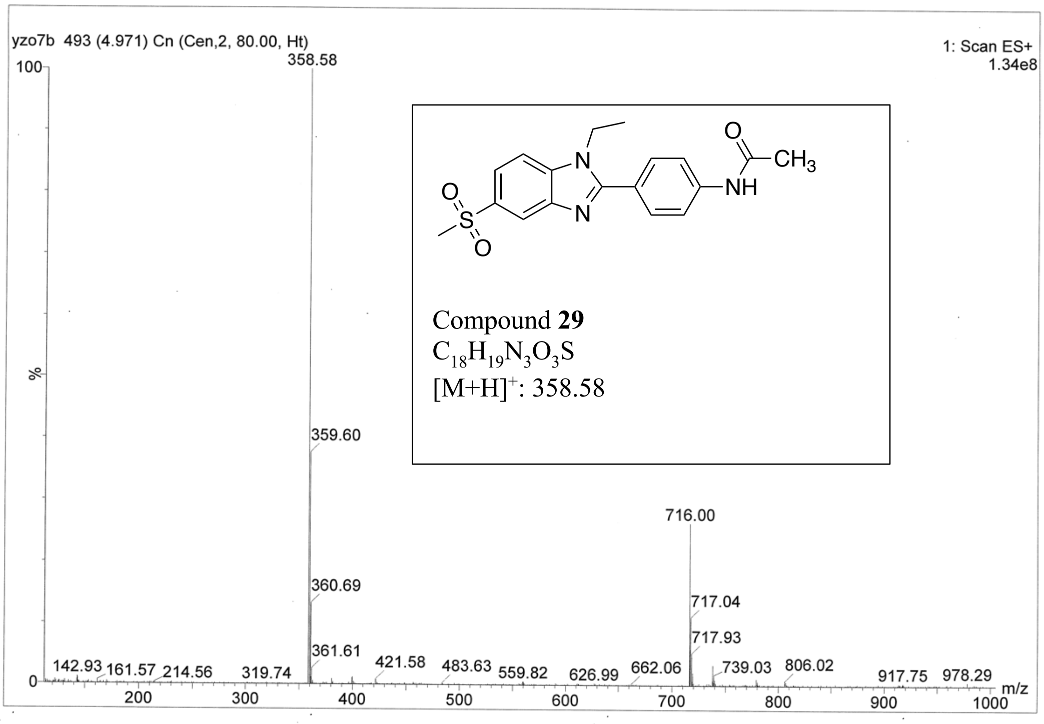


**Figure S15.** ESI-MS spectrum of compound **29**

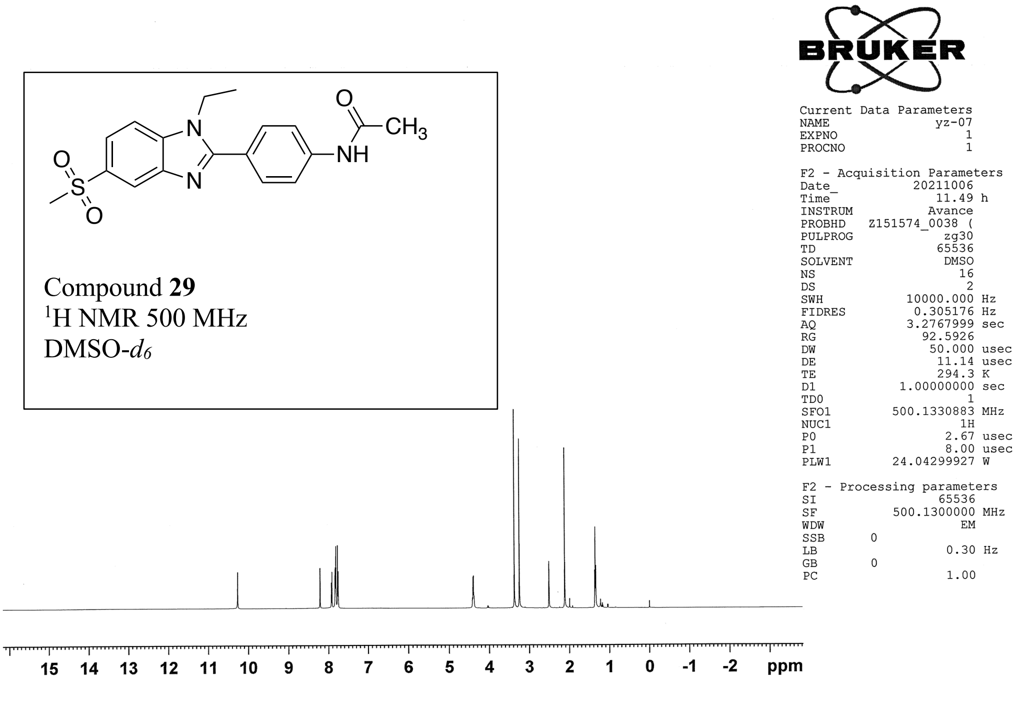


**Figure S16.** $^{1}H$ NMR spectrum of compound **29**

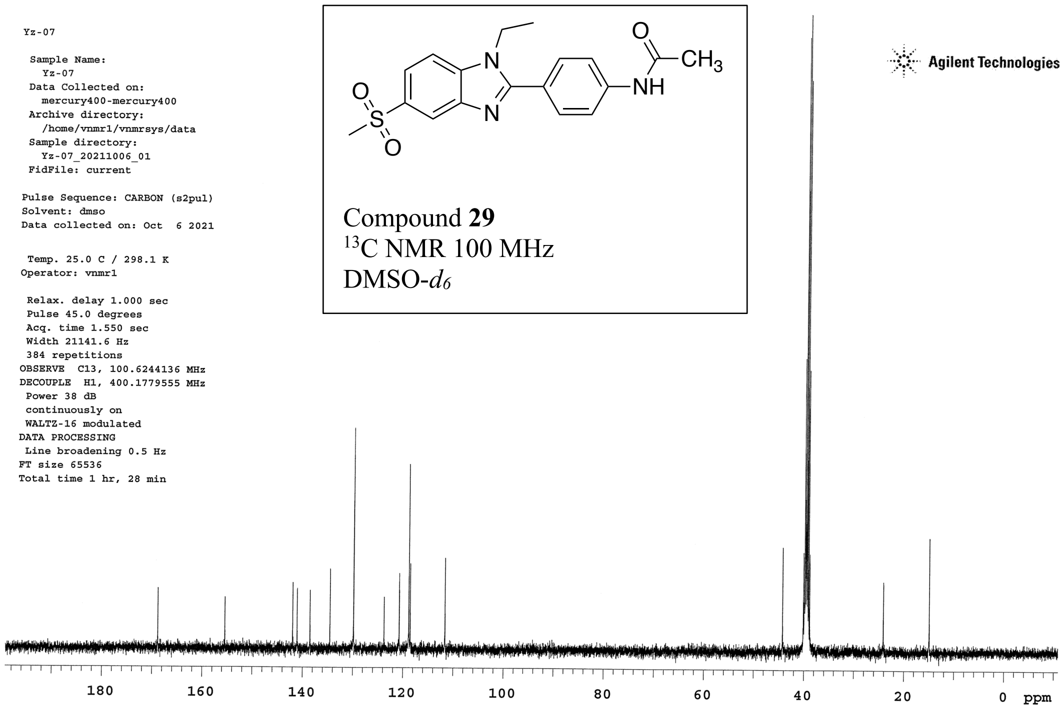


**Figure S17.** $^{13}$C NMR spectrum of compound **29**

1-Ethyl-2-(4-fluorophenyl)-5-(methylsulfonyl)–1*H*-benzo[*d*]imidazole (**30**)

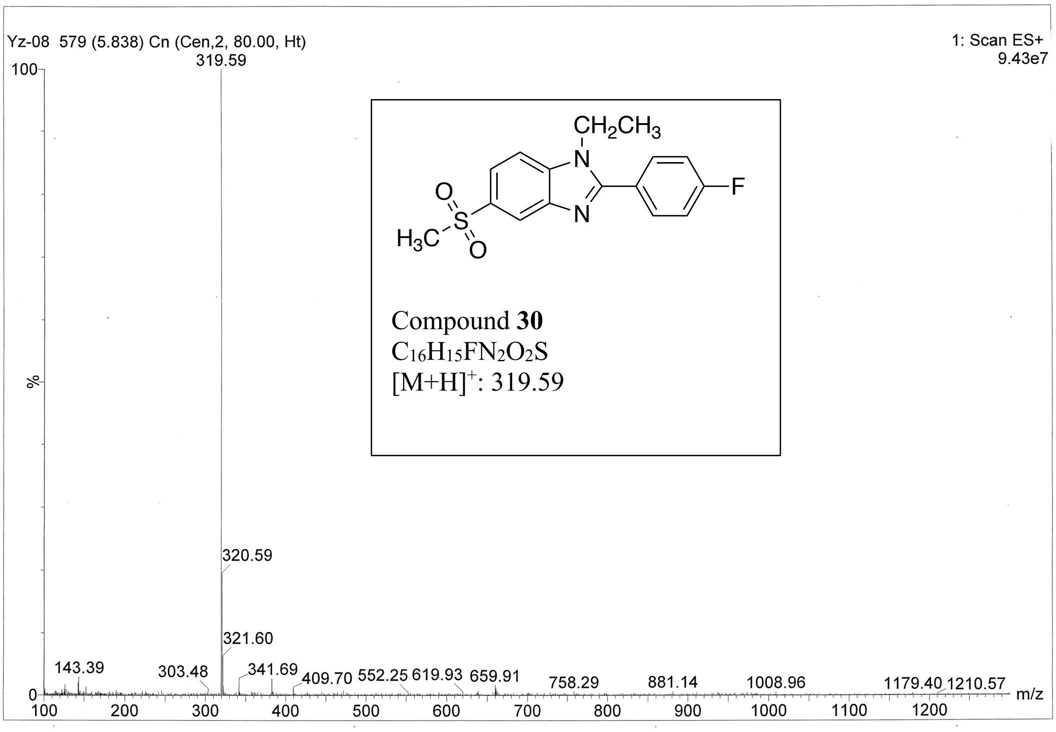


**Figure S18.** ESI-MS spectrum of compound **30**

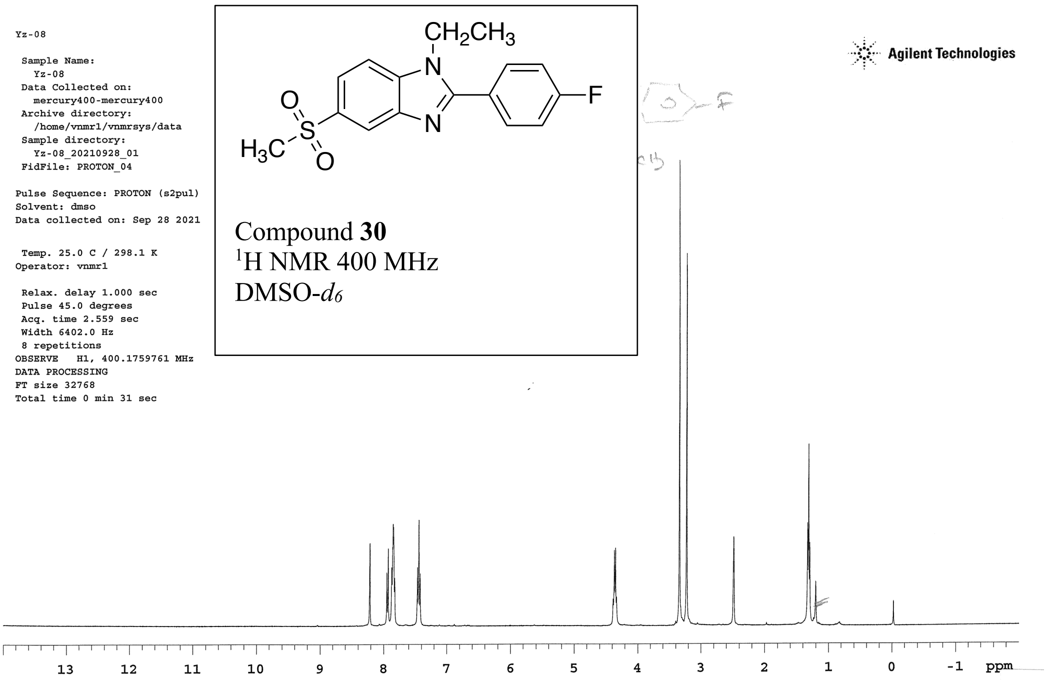


**Figure S19**. $^{1}$H NMR spectrum of compound **30**

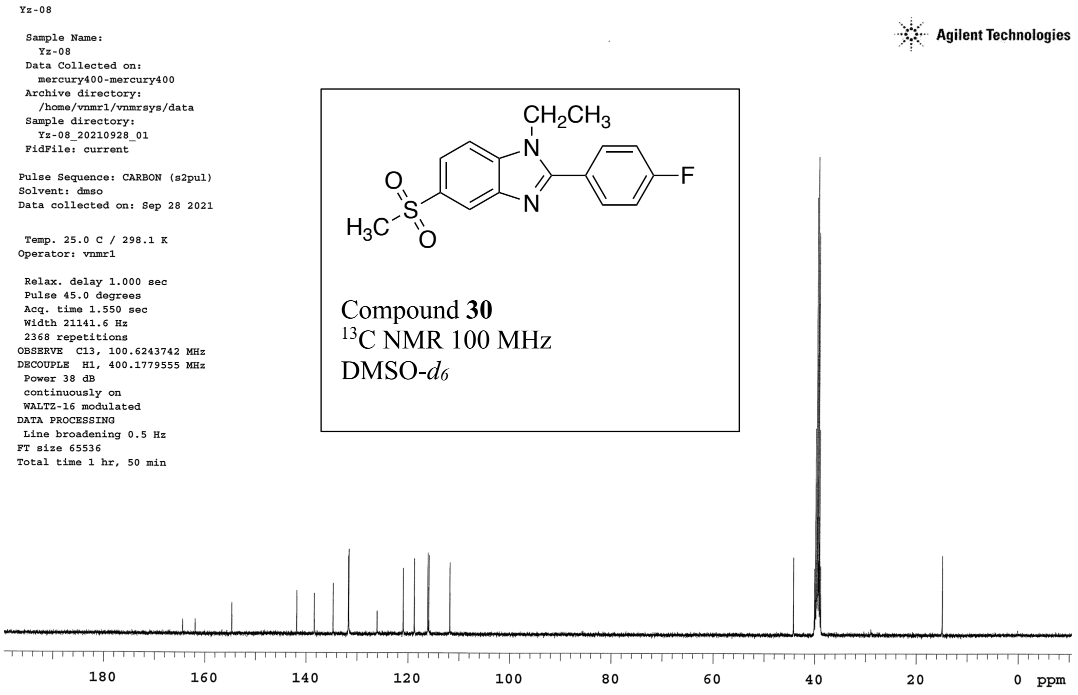


**Figure S20.** $^{13}$C NMR spectrum of compound **30**

1-Butyl-5-(ethylsulfonyl)-2-(4-fluorophenyl)–1*H*-benzo[*d*]imidazole (**31**)

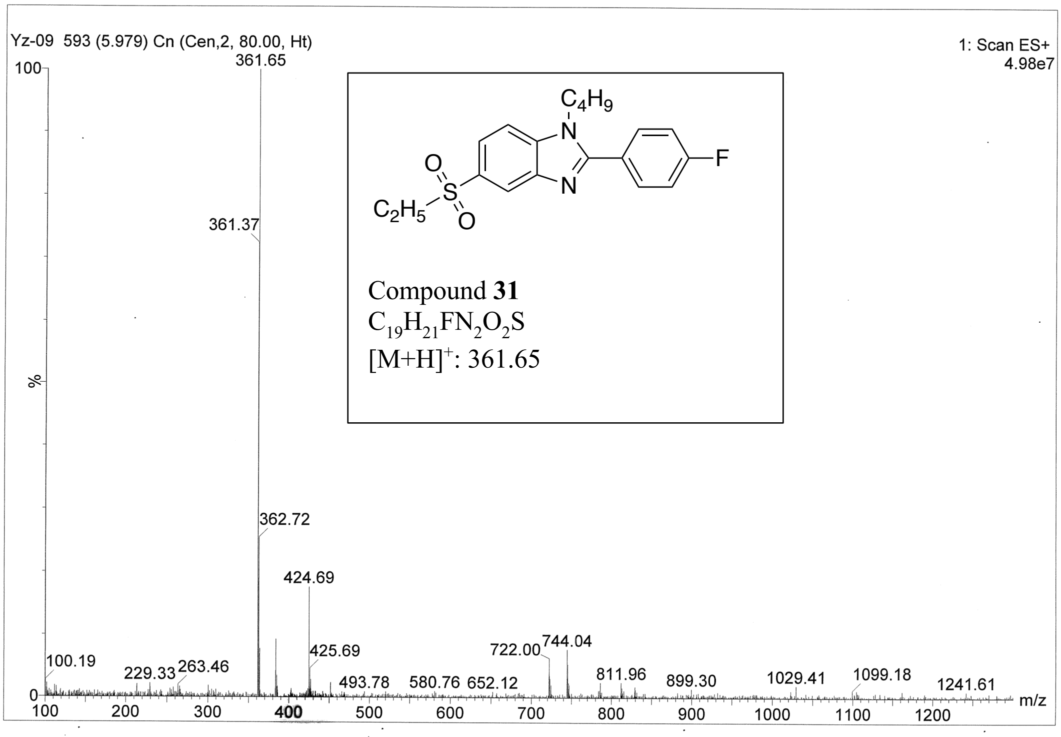


**Figure S21.** ESI-MS spectrum of compound **31**

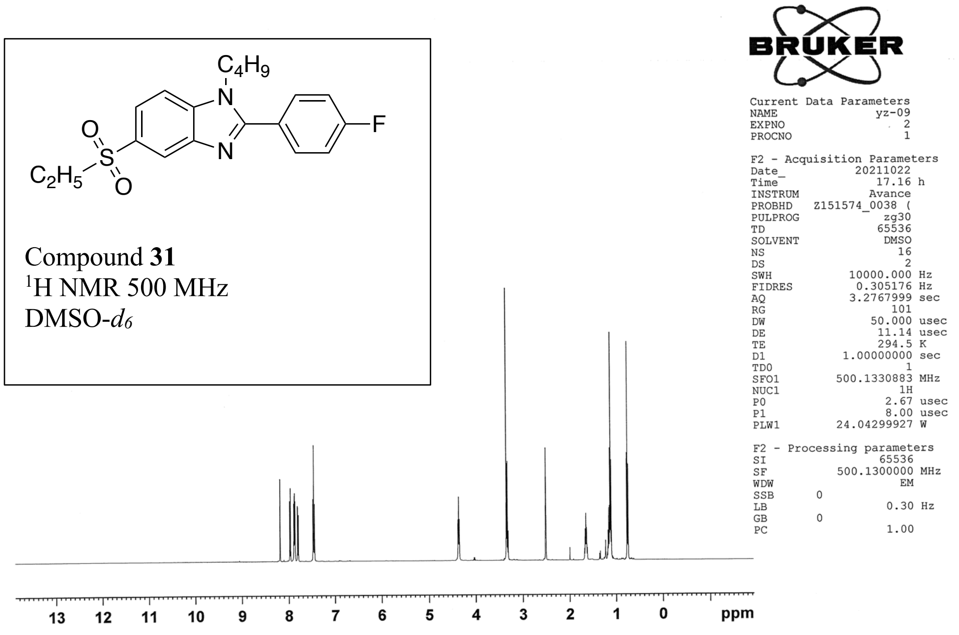


**Figure S22.** $^{1}H$ NMR spectrum of compound **31**

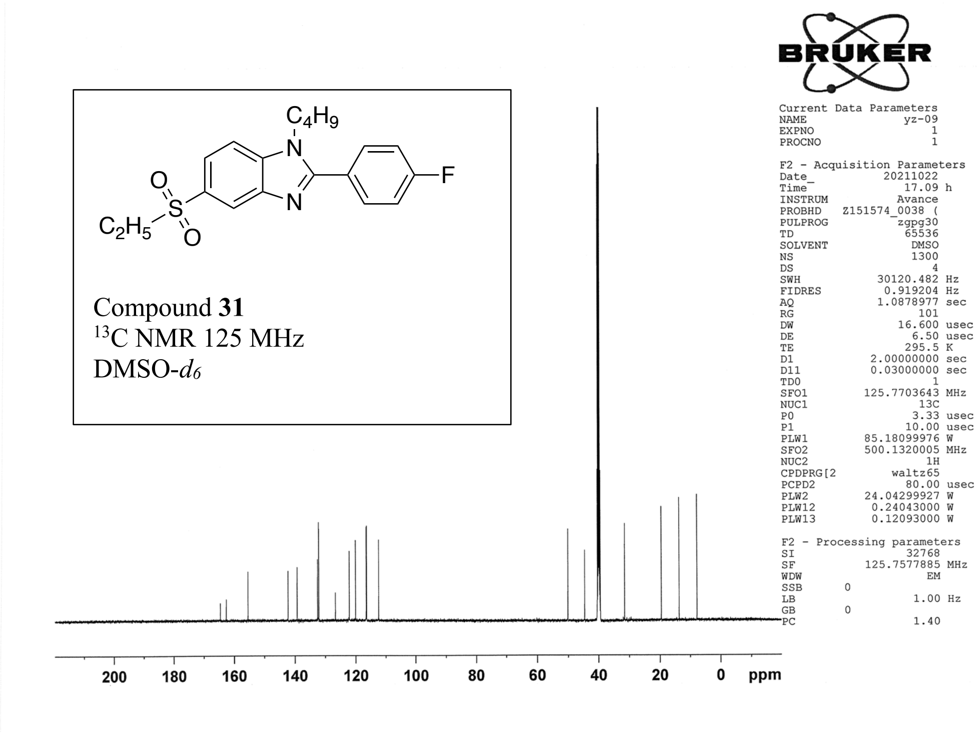


**Figure S23.** $^{13}C$ NMR spectrum of compound **31**

<u>*N*-(4-(1-Butyl-5-(ethylsulfonyl)-1*H*-benzo[*d*]imidazol-2-yl) phenyl)acetamide (**32**)</u>

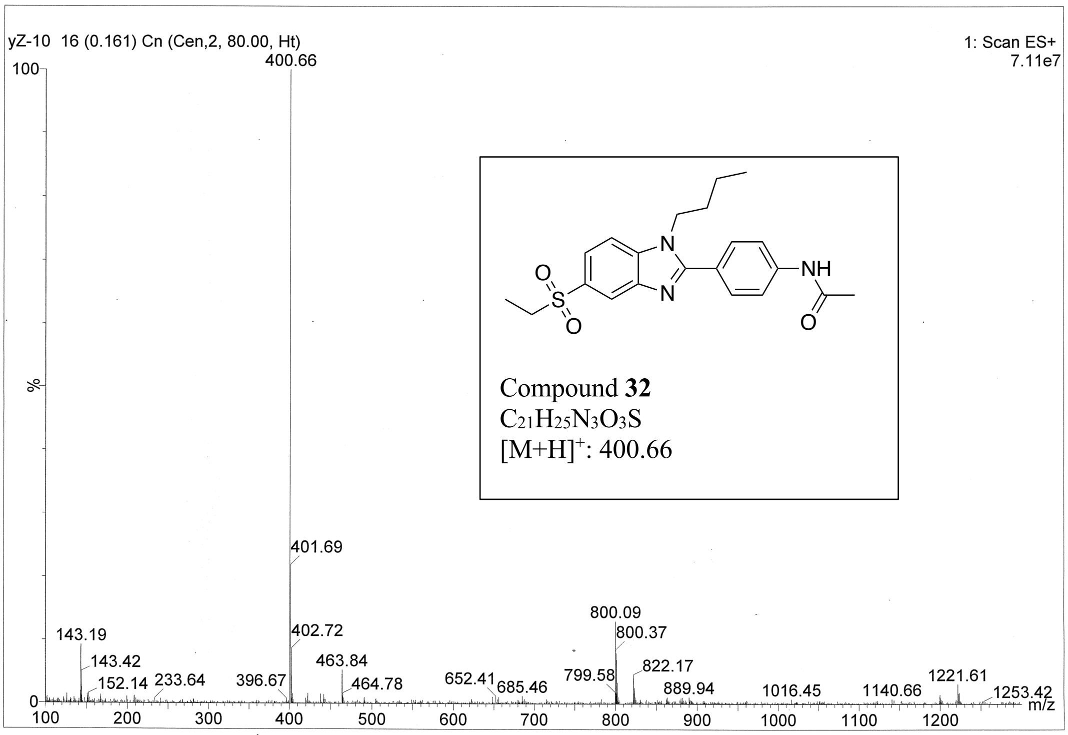


**Figure S24.** ESI-MS spectrum of compound **32**

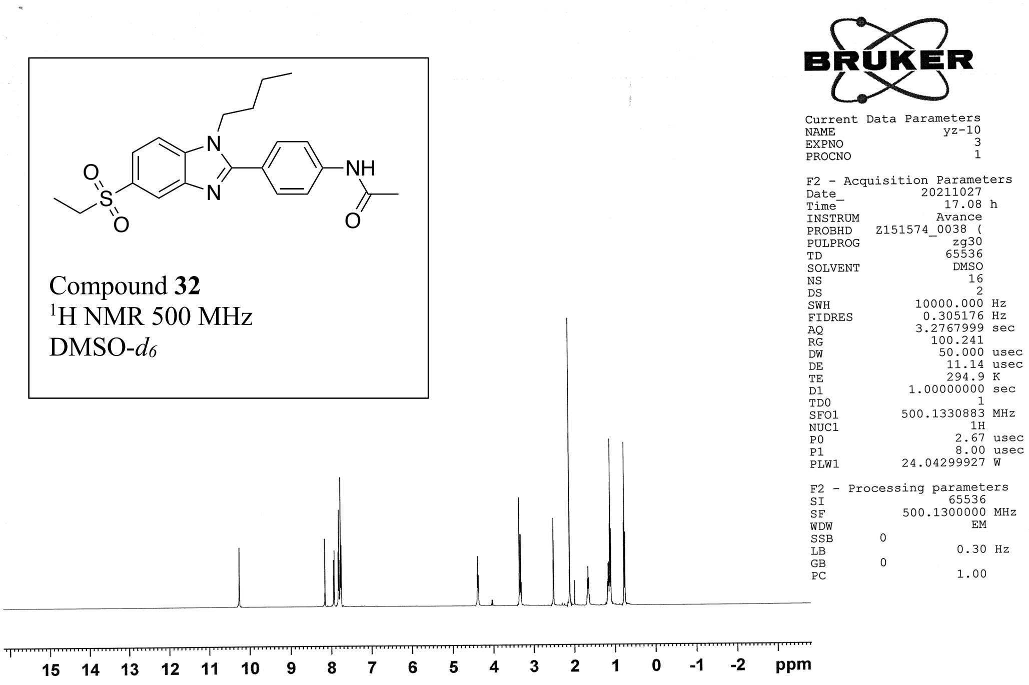


**Figure S25.** $^{1}$H NMR spectrum of compound **32**

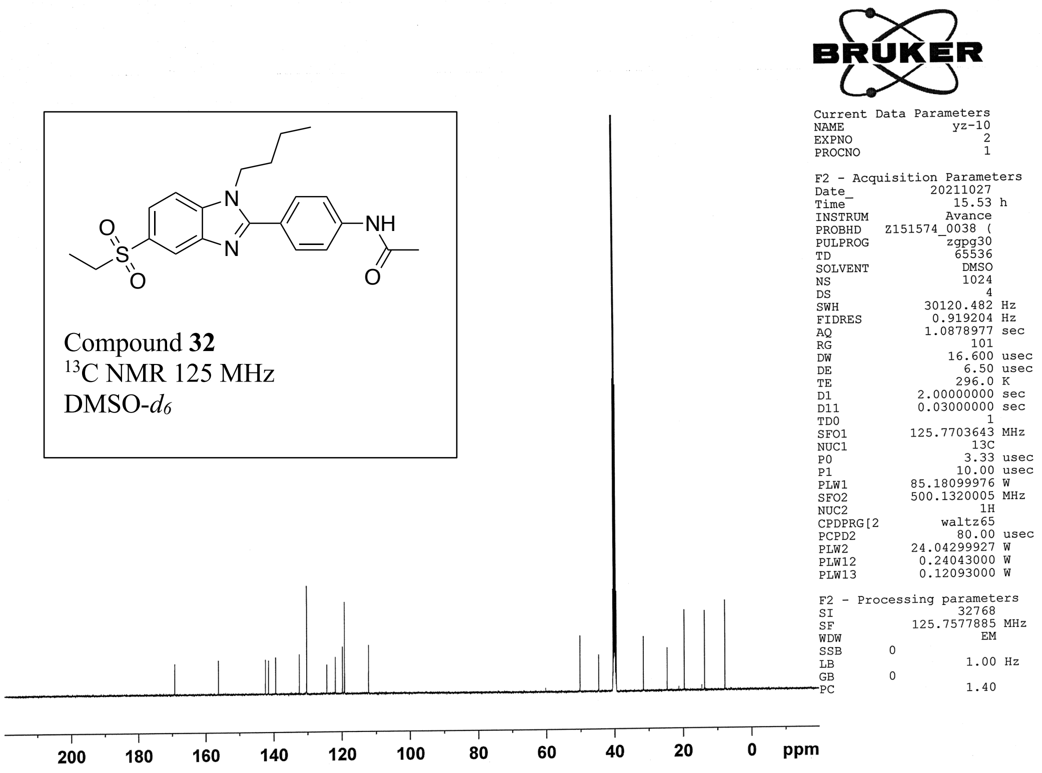


**Figure S26.** $^{13}$C NMR spectrum of compound **32**

Methyl 4-(1-(3,4-difluorobenzyl)-5-(methylsulfonyl)-1*H*-benzo[*d*]imidazol-2-yl)benzoate (**33**)

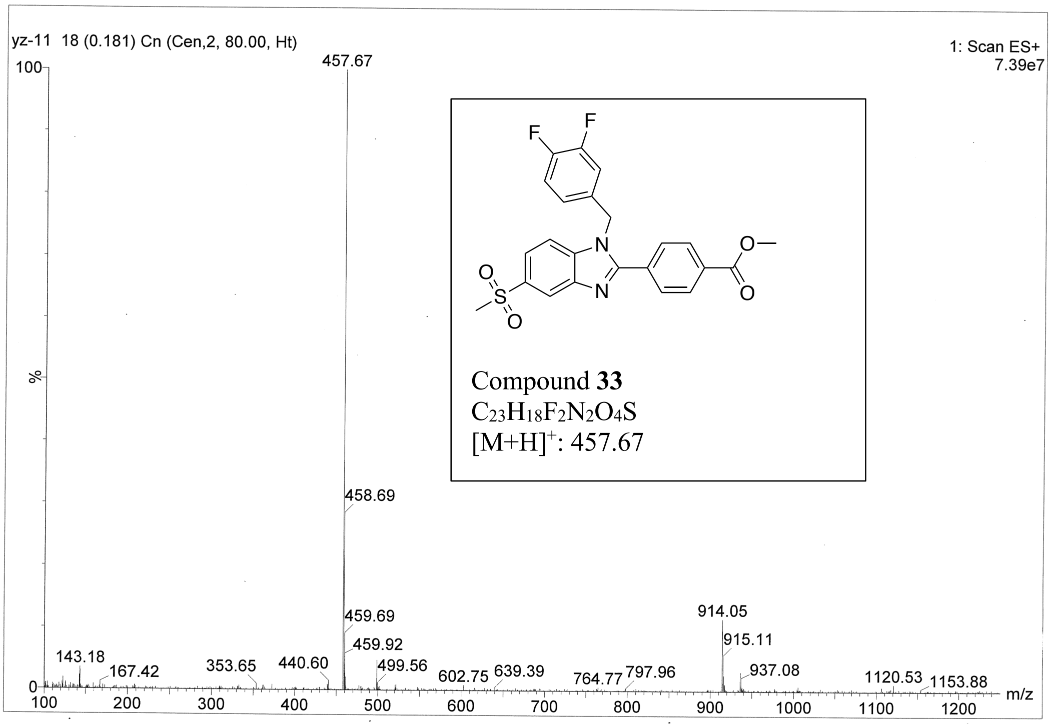


**Figure S27.** ESI-MS spectrum of compound **33**

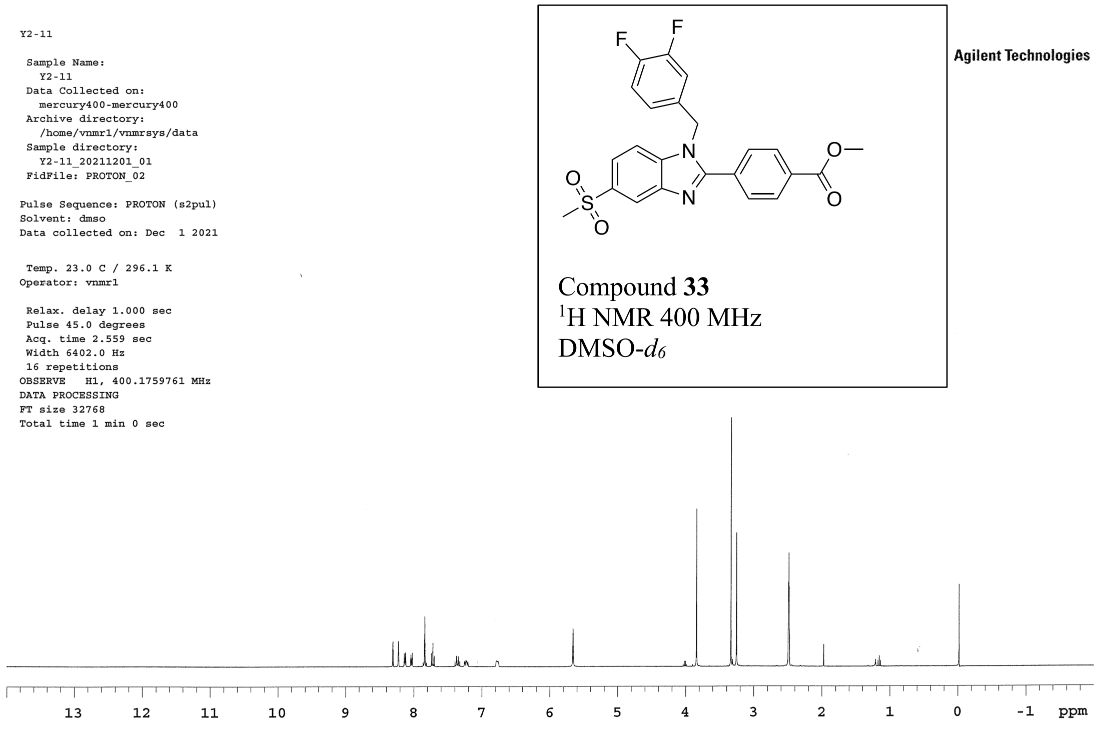


**Figure S28.** $^1$H NMR spectrum of compound **33**

2-(4-Chlorophenyl)-1-propyl-5-(propylsulfonyl)–1*H*-benzo[*d*]imidazole (**34**)

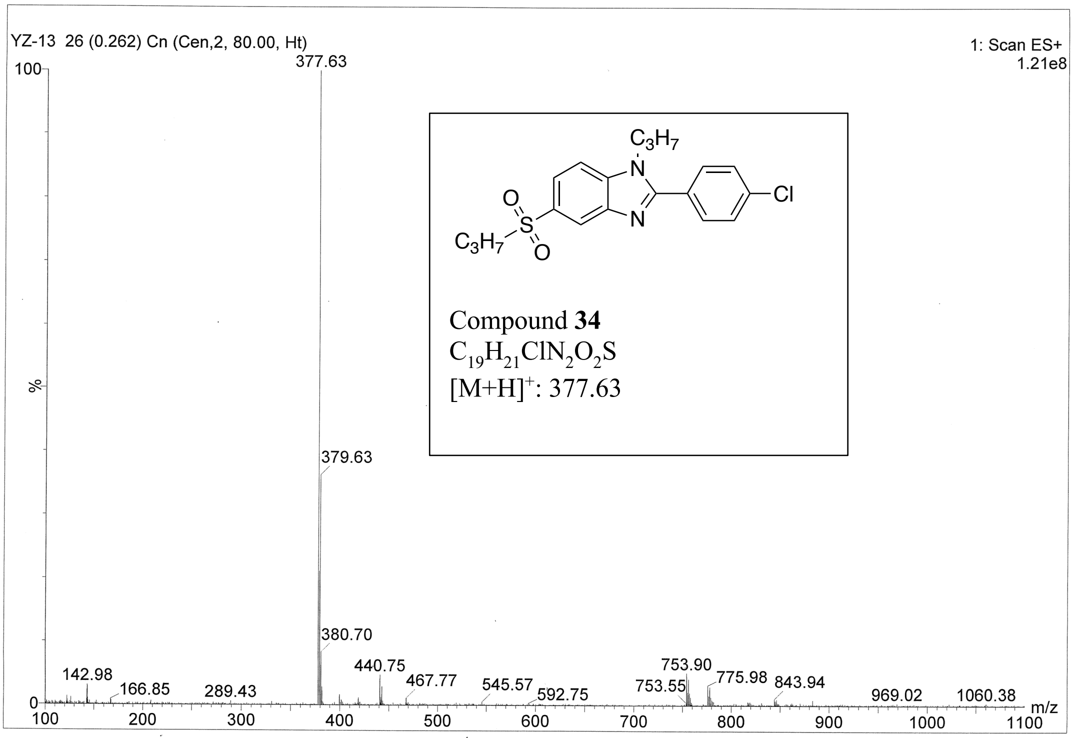


**Figure S29.** ESI-MS spectrum of compound **34**

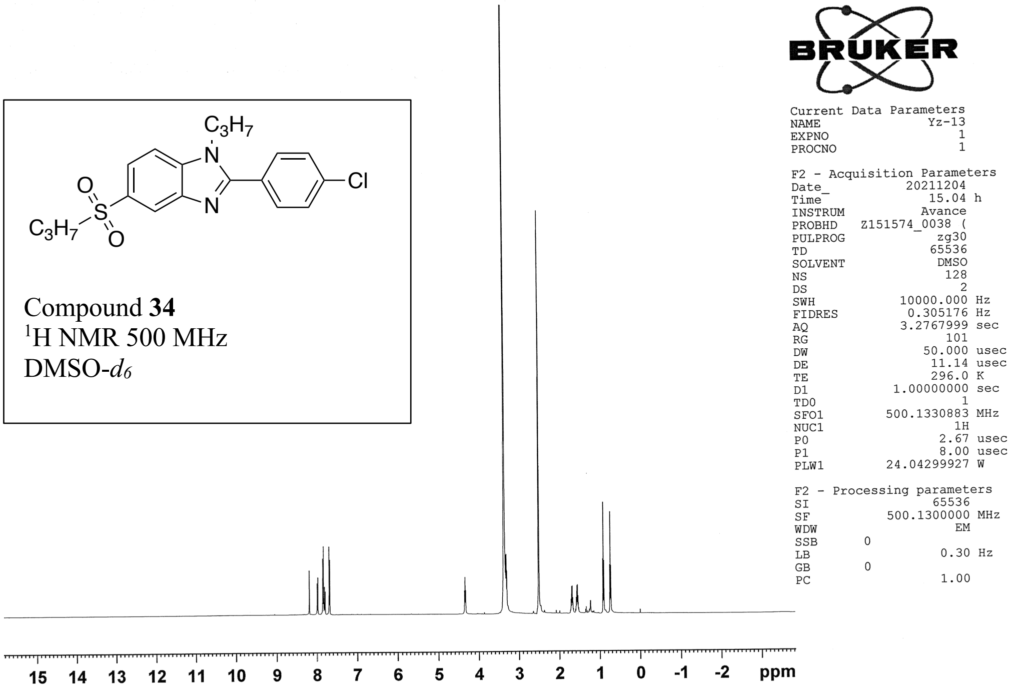


**Figure S30.** $^{1}$H NMR spectrum of compound **34**

2-(2,5-Difluorophenyl)-1-(4-fluorobenzyl)-5-(methylsulfonyl)–1*H*-benzo[*d*]imidazole (**35**)

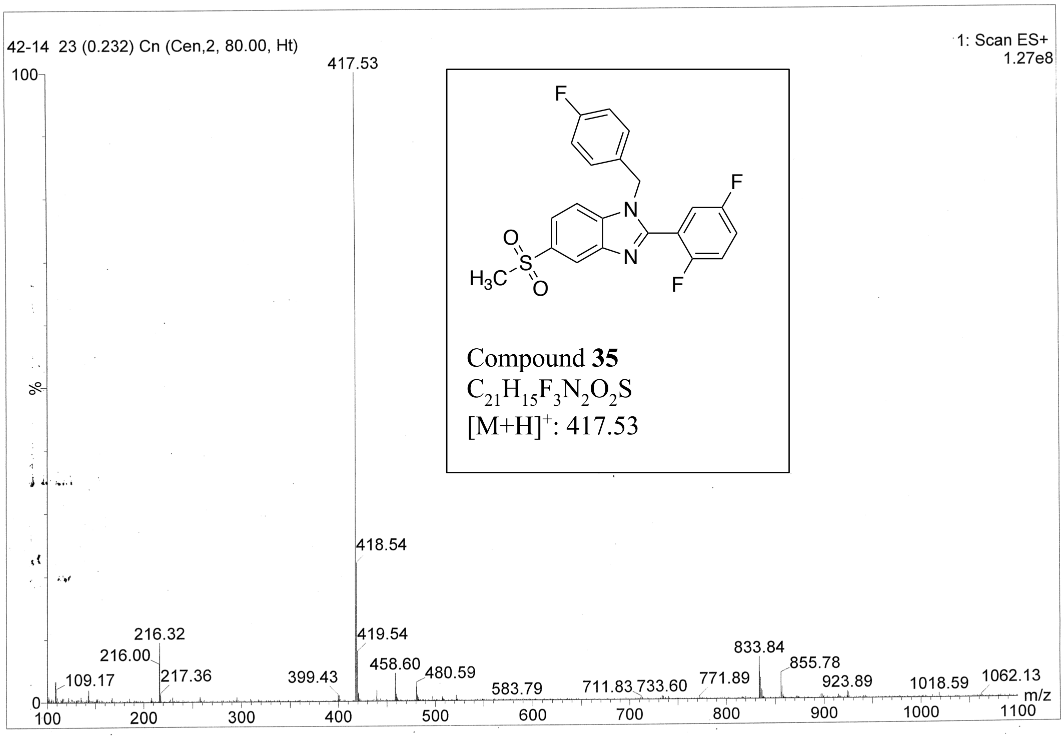


**Figure S31.** ESI-MS spectrum of compound **35**

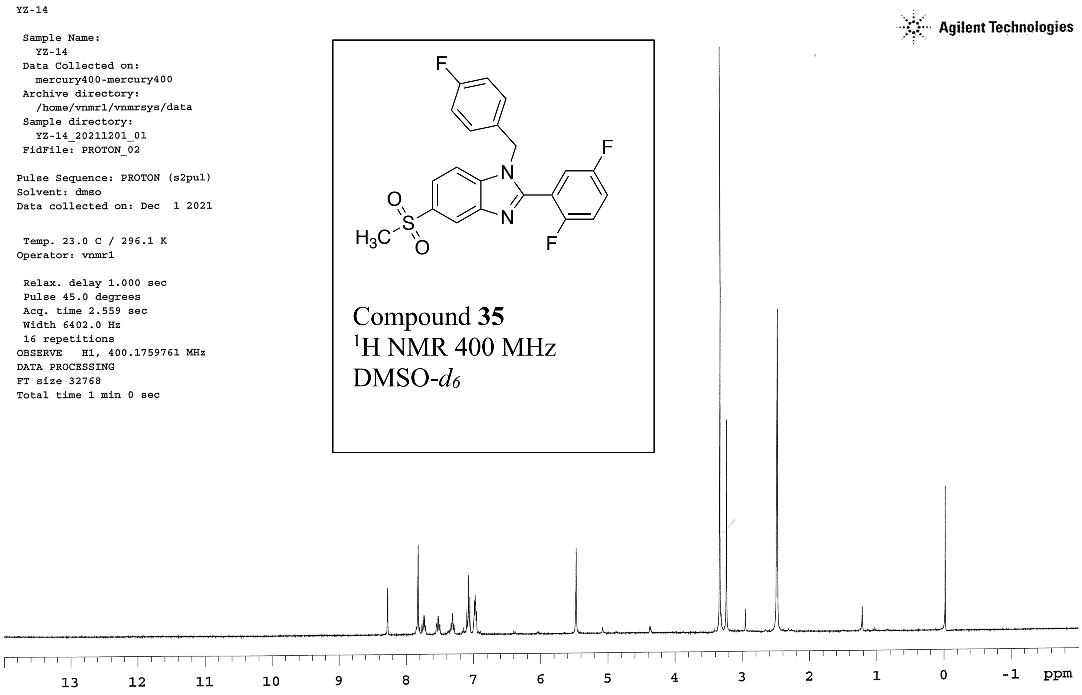


**Figure S32**. $^{1}$H NMR spectrum of compound **35**

<u>*N*-(4-(1-(4-fluorobenzyl)-5-(methylsulfonyl)-1*H*-benzo[*d*]imidazol-2-yl)phenyl)acetamide (**36**)</u>

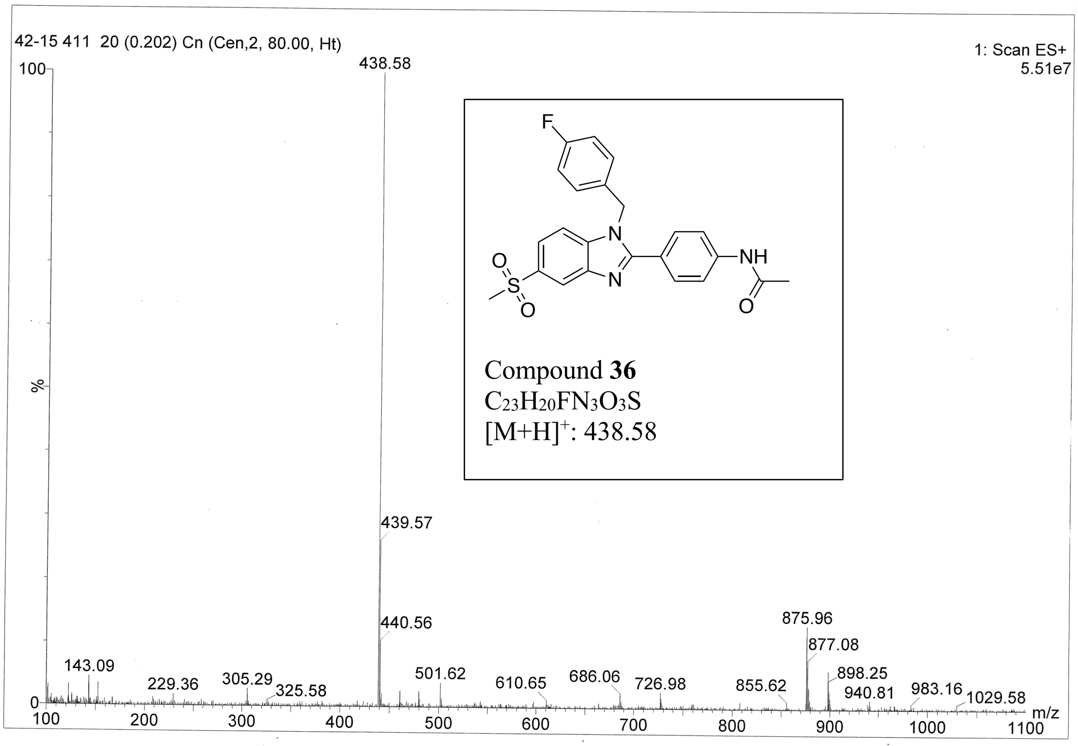


**Figure S33.** ESI-MS spectrum of compound **36**

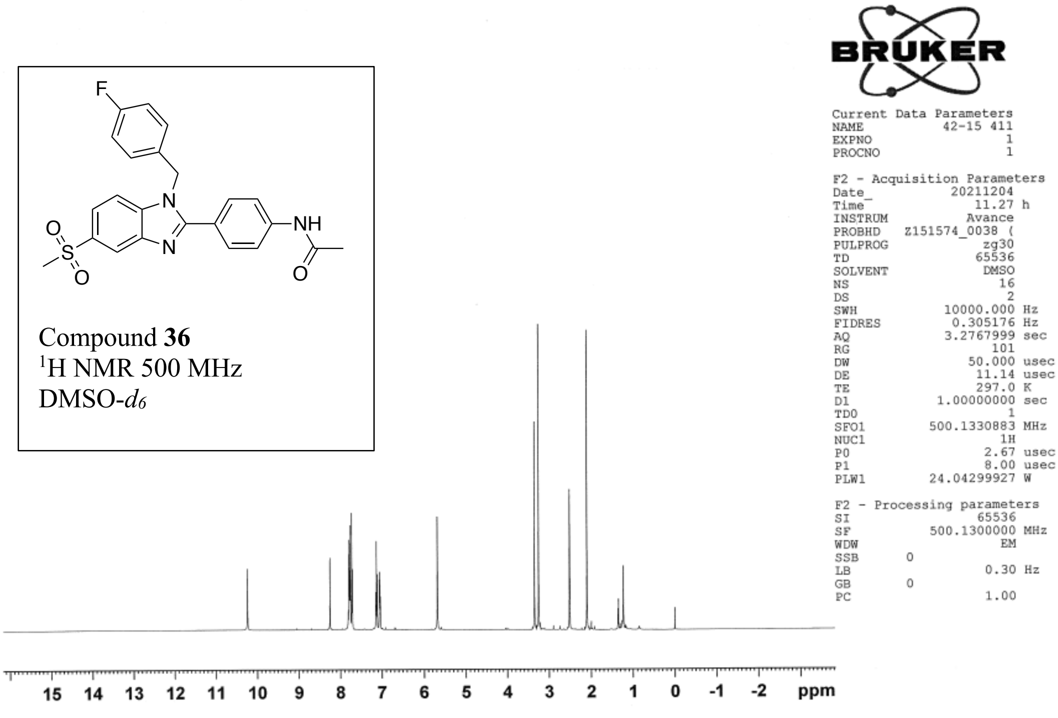


**Figure S34.** $^{1}$H NMR spectrum of compound **36**

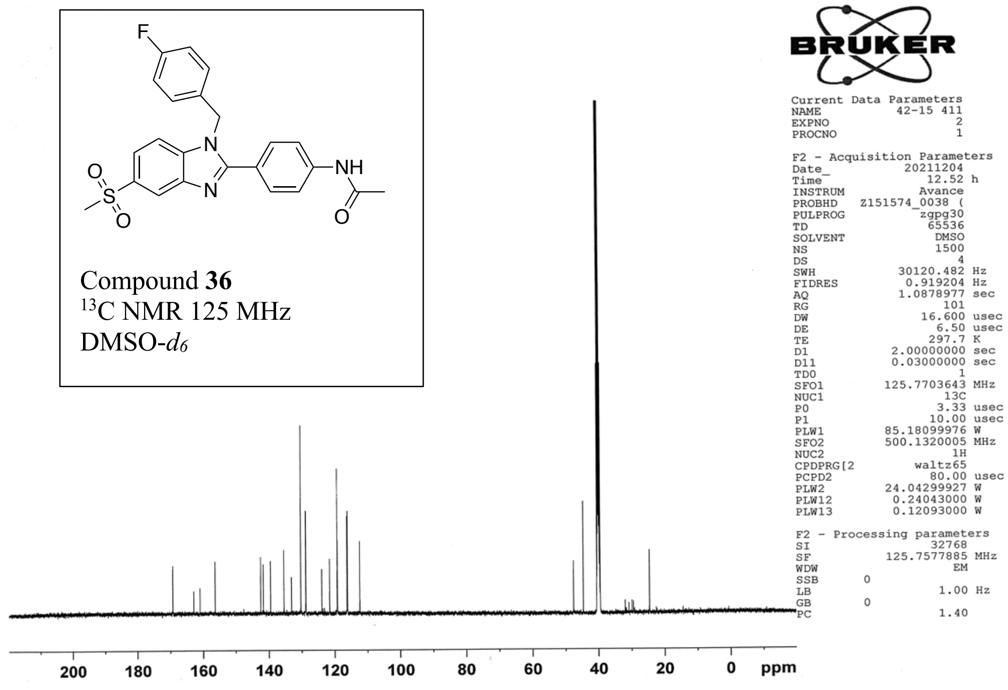


**Figure S35**. $^{13}C$ NMR spectrum of compound **36**

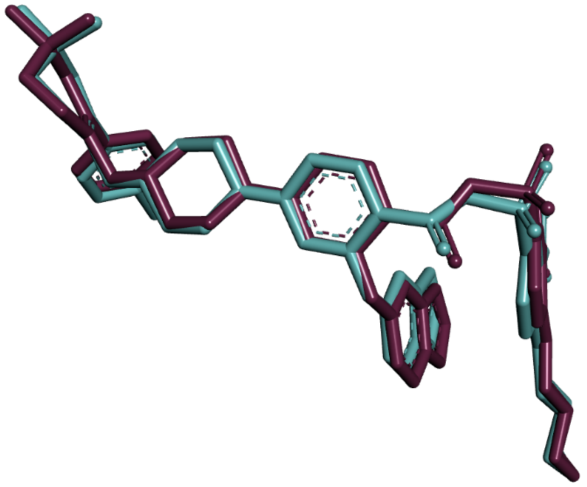

**Figure S36.** Alignment of Bcl-2 co-ligand venetoclax (dark red) and its re-docked conformer (turquoise). Root mean square deviation (RMSD) between the natural pose and docked poses is 0.574 Å.

**Table S1.** HOMO-LUMO gap values for compound **23-36**.

| Comp. | HOMO-LUMO Gap (eV) |
|---|---|
| **23** | 3.126 |
| **24** | 4.460 |
| **25** | 4.395 |
| **26** | 3.480 |
| **27** | 2.135 |
| **28** | 4.255 |
| **29** | 4.268 |
| **30** | 3.210 |
| **31** | 3.204 |
| **32** | 4.282 |
| **33** | 4.090 |
| **34** | 3.109 |
| **35** | 2.959 |
| **36** | 4.149 |
